# Results of the *Optimune* trial: A randomized controlled trial evaluating a novel Internet intervention for breast cancer survivors

**DOI:** 10.1371/journal.pone.0251276

**Published:** 2021-05-07

**Authors:** Franziska Holtdirk, Anja Mehnert, Mario Weiss, Johannes Mayer, Björn Meyer, Peter Bröde, Maren Claus, Carsten Watzl

**Affiliations:** 1 Research Department, Gaia Group, Hamburg, Germany; 2 Department of Medical Psychology and Medical Sociology University Hospital of Leipzig, Leipzig, Germany; 3 Leibniz Research Centre (IfADo), Technical University of Dortmund, Dortmund, Germany; Universidad Miguel Hernandez de Elche, SPAIN

## Abstract

**Introduction:**

After the acute treatment phase, breast cancer patients often experience low quality of life and impaired mental health, which could potentially be improved by offering cognitive behavioural therapy (CBT) and addressing exercise and dietary habits. However, CBT and other behavioural interventions are rarely available beyond the acute treatment phase. Internet-based interventions could bridge such treatment gaps, given their flexibility and scalability. In this randomized controlled trial (RCT), we investigated the effects of such an intervention (“*Optimune*”) over three months.

**Methods:**

This RCT included 363 female breast cancer survivors (age range = 30–70), recruited from the community, who had completed the active treatment phase. Inclusion criteria were: breast cancer diagnosis less than 5 years ago and acute treatment completion at least 1 month ago. Participants were randomly assigned to (1) an intervention group (*n* = 181), in which they received care as usual (CAU) plus 12-month access to *Optimune* immediately after randomization, or (2) a control group (*n* = 182), in which they received CAU and *Optimune* after a delay of 3 months. Primary endpoints were quality of life (QoL), physical activity, and dietary habits at three months. We hypothesized that intervention group participants would report better QoL, more physical activity, and improved dietary habits after 3 months.

**Results:**

Intention-to-treat (ITT) analyses revealed significant effects on QoL (*d* = 0.27, 95% CI: 0.07–0.48) and dietary habits (*d* = 0.36, 95% CI: 0.15–0.56), but the effect on physical exercise was not significant (*d* = 0.30; 95% CI: 0.10–0.51).

**Discussion:**

These findings suggest the effectiveness of *Optimune*, a new CBT-based Internet intervention for breast cancer survivors, in facilitating improvements in quality of life and dietary habits. Efforts to disseminate this intervention more broadly may be warranted.

**Trial registration:**

ClinicalTrials.gov, NCT03643640. Registered August 23rd 2018, https://clinicaltrials.gov/ct2/show/NCT03643640.

## Introduction

With an incidence of 2.09 million women each year, breast cancer is the most frequent type of cancer among women worldwide [1]. After the initial acute treatment phase, which often includes surgery, radiation and aggressive combination-chemotherapy, patients typically progress to a monitoring phase, during which they may receive lower intensity treatments with anti-hormones, aromatase-inhibitors or bisphosphonates [2]. Psychological support is often offered during the acute treatment phase but is rarely available in monitoring phases, even though between 25% and 52% of breast cancer survivors experience clinically significant distress, including fatigue, depression, anxiety and impaired quality of life, even years after acute phase treatment [3–7]. Moreover, psychological distress may be linked with chronic systemic inflammation, which in itself could be a predictor of a worse prognosis over time [8–10].

Both pharmacological treatments and psychological interventions, such as Cognitive Behaviour Therapy (CBT) and mindfulness meditation, could be used to treat clinically relevant depression or anxiety and potentially reduce inflammation among breast cancer survivors [11–20]. Of note, meta-analytic evidence suggests that antidepressants are prescribed for as many as 23% of breast cancer survivors, despite some controversy as to whether selective serotonin re-uptake inhibitors (SSRI) interact with tamoxifen [21, 22]. There is some evidence that certain SSRI might alleviate depression because of their effects on cytokines such as IL-6 and TNF-α [20]. Conversely, it has also been observed that targeting inflammation with NSAIDs and cytokine-inhibitors could alleviate depressive symptoms [19].

Psychological interventions have also been found to improve well-being and potentially reduce inflammation. For example, mindfulness-based stress reduction (MBSR), CBT and supportive-expressive dynamic psychotherapy appear to reduce inflammatory markers (e.g., IL-6, TNF-α) in several diseases with comorbid depression [17, 18, 23]. A meta-analysis reported that mindfulness meditation reduces inflammatory biomarkers such as CRP or NF-κB but may be neutral with regard to other cytokines [16]. One trial also found that a Yoga intervention reduced both depressive symptoms and markers of inflammation [15]. Studies with cancer survivors also suggest that psychological interventions such as CBT and mindfulness-based treatment improve quality of life while reducing inflammation [12–14].

Even though CBT and other psychological interventions could be beneficial for breast cancer survivors in the monitoring phase, a major challenge is that systematic survivorship programmes including psycho-oncological treatments are not always available, particularly in rural areas [24]. Barriers such as stigma concerns, scepticism regarding psychotherapy, time constraints, disease-related restrictions, lack of motivation, and perceived lack of necessity can also prevent patients from accessing these treatments [25]. Internet-based psychological interventions are often regarded as promising in this context because they could potentially deliver effective support in a flexible and efficient manner [26, 27]. Many randomized controlled trials (RCT) have demonstrated the efficacy of Internet interventions for a broad spectrum of mental health conditions as well as for illness management and health-related behaviour change [28–30]. However, there are also trials showing that some interventions are less effective than others, and some may even be harmful [31, 32]. Thus, it is necessary to examine the effects of each specific Internet intervention in methodologically adequate trials [30, 33].

As described in the study protocol [34], we developed an Internet intervention to provide psychological support for breast cancer survivors, named *Optimune*. This intervention was developed with the same technology and by the same multidisciplinary team that has developed a range of other evidence-based Internet interventions for conditions such as depression, anxiety disorders, harmful drinking, and psychological support for patients with multiple sclerosis and epilepsy [35–41]. *Optimune* contains 16 modules spanning topics such as stress management, emotion regulation, healthy dieting and regular exercise after breast cancer treatment, sleep management, and CBT techniques to improve mental health (see Methods section for a more detailed description). Given this broad range of content, *Optimune* can be described as a CBT-based, holistic Internet intervention. *Optimune* aims to engage users in a broad array of CBT methods and behaviour change techniques (see intervention description below) in order to improve their quality of life and to facilitate the likelihood that they adopt habits that support their immune health, particularly dietary and exercise habits. By engaging in these exercises, users could potentially learn how to manage or reduce stress and how to adopt and maintain healthier dietary and exercise habits.

The goal of this pragmatic RCT was to test effects of *Optimune* over the course of three months on quality of life (QoL) and on two relevant health behaviours: dietary habits and physical exercise (primary endpoints). Trial success was defined a priori [34] as showing a significant effect on at least one of these endpoints at 3 months because improvements in some modifiable lifestyle factors are better than none, and even partial improvements in health behaviours or quality of life could be valuable outcomes in their own right, which might lead to improvements in immune function or disease prognosis. Secondary outcomes included insomnia, fear of cancer recurrence, cancer-related emotional stress, depression and anxiety symptoms. We also examined the subjective utility of the intervention as well as potential adverse effects.

The intervention is intended to be used adjunctively to other treatments patients may receive; therefore, in this trial it was offered in addition to care-as-usual (CAU). The relevant control group was CAU-only because we were interested in examining intervention effects under routine care conditions [42, 43]. The primary hypothesis was that participants randomized to the intervention, compared to those in the control condition, would have better quality of life as well as healthier dietary habits and physical exercise habits at 3 months. To examine the stability of effects, we also collected data at a follow-up time-point 6 months after baseline.

## Methods

The ethics committee of the IfADo—Leibniz-Institut für Arbeitsforschung at the TU (Technical University) Dortmund, Germany, approved of the study.

### Study design

In this parallel-groups RCT, participants were randomly assigned to (1) the intervention group (IG), in which they immediately received access to *Optimune*, on top of CAU, or (2) the control group (CG), in which they received CAU only. Data were collected at baseline, 3 months (time-point of primary interest), and 6 months post-baseline (follow-up). Participants in the CG were offered access to *Optimune* after 3 months; therefore, the CG is a combination of CAU and wait-list (CAU/WL). Participants were not blinded to group assignment, given the design of the intervention. Simple randomization (no blocks or stratification) was performed by the Principal Investigator with a computer-generated sequence. Concealed allocation to conditions was ensured because research team members who informed participants of the randomization result did not have access to any participant data and were not informed of the randomization result until after inclusion of a participant.

### Recruitment and assessment

Participants were recruited in Germany from a broad range of settings, including Internet advertisements, treatment clinics, patient associations, support groups, and health insurance companies. Potentially interested women were referred to the study homepage, where they could sign up with their name and email address to request further information. Potential participants then received an email invitation with detailed information about the study and an invitation to an online baseline assessment. All participants were required to provide online informed consent, and those who were potentially eligible had to provide a discharge letter from their oncology treatment centre or clinic in order to verify diagnoses and therapies. Final inclusion into the study occurred after research team members had confirmed receipt of such a letter and had checked all inclusion and exclusion criteria. Randomization took place immediately after inclusion. In case participants assigned to the IG failed to register to *Optimune* within one week after randomization, they were contacted by the blinded technical support team. Email invitations were sent for the 3 and 6 months assessments, and up to two reminders were sent to those who did not respond to the initial invitation.

### Inclusion criteria

Inclusion criteria were female sex, having received a breast cancer diagnosis less than 5 years ago, and having completed acute treatment for breast cancer (such as surgery, chemotherapy or radiation treatment) at least 1 month prior to study entry. Concurrent treatment with anti-hormone therapy such as tamoxifen, aromatase-inhibitors or bisphosphonates was permissible. Participants were required to be between 30 and 70 years of age, to provide informed consent, to be able to speak and read German, and to provide treatment discharge letter to verify diagnoses and treatments.

### Intervention: *Optimune*

The CBT-based, holistic Internet-based intervention *Optimune* was developed by a team of clinical psychologists, CBT therapists, physicians, software engineers, graphic artists, and professional speakers, among others, affiliated with GAIA in Hamburg, Germany. GAIA is an SME (small to medium enterprise) specialized on the development of digital treatment programmes, with a track record of more than 20 such programmes, many of which have been evaluated in independent RCTs [35–41, 44]. *Optimune* is based primarily on established CBT techniques targeting depression, anxiety, and fatigue. The intervention also engages users in therapeutic techniques that have been shown to have beneficial effects on immune system functioning and inflammation, including sleep and stress management (e.g., mindfulness-based techniques) and health behaviour change (dietary habits and physical activity advice). The dimensions addressed in the programme are consistent with the German treatment guidelines for breast cancer survivors. The programme also includes a total of 240 citations to relevant scientific literature, with patient-friendly summaries (annotated bibliography). All therapeutic techniques are conveyed via the format of a “simulated dialogue” in which users read information or listen to audio clips and then have to choose one of several predefined response options. Subsequent content is continuously tailored based on these user responses. The programme also contains illustrations and photographs, pdf-summaries, and daily text messages that are intended to remind and motivate patients to use the programme. *Optimune* was developed with a responsive web-design approach and can be accessed via a password-protected, secure (https-encrypted) website (https://optimune.broca.io) with any computer or smartphone equipped with a contemporary web browser. Patient and expert feedback was sought continuously during the development process, and content as well as functions were iteratively refined based on this feedback. The intervention was produced on a proprietary software platform (broca^®^) developed by GAIA. It uses cloud computing with fast global access and is securely hosted in an ISO-27001-certified data centre located in Germany.

After registration with a personal 12-digit code, users can access *Optimune* for a period of one year, even though it is assumed that they can complete the programme much sooner. A total of up to 16 modules is offered, although there is no expectation or requirement for users to finish all modules. There is no fixed or generic sequence in which modules must be completed. The intervention uses algorithm-driven sequences to guide users through the programme, and they are invited to flexibly explore the content whenever they wish. A general recommendation offered to users is to complete at least 1 to 2 sessions of around 30 minutes per week. Module length varies depending on factors such as reading speed, individual path taken through the module, desire to explore greater or lesser depth, and decisions to listen or not listen to optional audio clips. Users are also encouraged to discontinue using the intervention if they feel that it is not helpful.

The 16 modules can be grouped into four content domains: (1) psychological well-being, (2) dietary coaching, (3) physical activity and exercise, and (4) sleep management. Functions, purpose and time-frame of the intervention are covered in an introductory module. Subsequent modules cover some psychoeducational content but primarily CBT-based exercises to enhance emotion regulation, improve well-being, ward off depression and anxiety, optimize coping with common concerns and hassles associated with breast cancer and its treatment, adhere to a healthy diet, exercise regularly, and use CBT techniques to overcome insomnia. Evidence-based behaviour change techniques such as goal setting, action planning, mental contrasting (of pros and cons or goal attainment obstacles), mental imagery exercises, case examples to encourage modelling, problem-solving, rehearsing implementation intentions, and positive self-statements are used in every module. Dimensions used to custom-tailor content include variables such as current stress level, dietary habits (e.g., vegetarian diet), or fitness and exercise habits and preferences. A more detailed description of the functions and content of *Optimune* is provided in the study protocol [34].

### Outcome measures

#### Primary endpoints

The primary endpoints were (1) overall quality of life, measured with the total score of the World Health Organization Quality of Life Questionnaire (WHOQOL-BREF, 26 items) [45]); (2) physical exercise, measured with the total score of the International Physical Activity Questionnaire (IPAQ, 27 items) [46]); and (3) dietary habits, measured with the total score of the Food Quality Questionnaire (FQQ, 20 items, see section below for further details). Trial success was defined a priori as demonstrating a significant intervention effect on at least one of these three primary endpoints [34].

*Development of the FQQ*. Whereas the WHOQOL-BREF and the IPAQ are well validated questionnaires, the FQQ was developed for this trial as a face-valid self-report measure of current dietary habits, with an emphasis on selected foods that are thought to either facilitate or reduce inflammation. The FQQ items were developed based on consensus discussions among the research team members, several of whom were involved in the development of *Optimune*. Two main criteria had to be met in order for an item to be included: (a) There had to be agreement that the respective food item was regarded as clearly healthy or unhealthy, according to information presented in *Optimune* and in line with current evidence [47–49], and (b) each food item had to be common and easily available throughout Germany. Food items for which there was controversy or lack of clear consensus as to whether they can be considered healthy or unhealthy in terms of their effects on inflammation were excluded (e.g., dairy products, red wine, meat). After review of potential alternative questionnaires with established validity, the research team concluded that no current questionnaire met these criteria, which justified the development of the FQQ.

The FQQ contains two 10-item subscales: (1) healthy foods and (2) unhealthy foods. Instructions are: “I eat (or drink/consume)”. Respondents are asked to indicate on a 4-point Likert-type scale how often they typically eat each of 20 items, which are presented in pseudo-random order. Response options are (0) very rarely, (1) rather rarely, (2) rather often, and (3) very often. This study represents an initial effort to examine the reliability and validity of the FQQ; internal consistency and factor structure are presented in the results section. The German version of the FQQ is available upon request from the authors and can be used freely by others.

The 10 items composing the FQQ healthy foods subscale are: (1) Kernels and seeds (e.g. sesame, linseed, pine and sunflower seeds), (2) Pome fruit (e.g. apple, pear, quince), (3) Stone fruit (e.g. apricot, peach, cherry, olive, plum), (4) Berries (e.g. raspberry, blackberry, blueberry, currant), (5) Fruit vegetables (e.g. cucumber, zucchini, tomato, bell pepper), (6) Onion vegetables (e.g. onion, garlic, leek), (7) Dried or fresh herbs (e.g. parsley, coriander, rosemary, thyme, dill), (8) Fresh or powdered spices (e.g. pepper, cinnamon, ginger, turmeric, cumin), (9) Olive oil, (10) Fatty fish (e.g. herring, sprat, salmon, mackerel, tuna).

The 10 items composing the FQQ unhealthy foods subscale are: (1) White bread or white flour buns, (2) Cookies or biscuits, (3) Pastries or sweet cakes (e.g. muffins, poppy seed snails, almond croissants) (4) Milk chocolate or chocolate bars, (5) Candy or sweets (e.g. gummy bears, hard candy, liquorice, marshmallows), (6) Processed or pre-packaged foods (e.g. instant soups, canned foods, microwave dishes, pre-packaged dishes), (7) Hamburgers or cheeseburgers, (8) White-flour pasta dishes, (9) French fries (chips), (10) Sweetened drinks (e.g. lemonade, cola, sweetened fruit spritzer).

In addition to computing these two subscales, we also constructed a total FQQ score which constituted one of the three primary endpoints. For the total FQQ score, all items of the unhealthy food subscale were reverse-scored, such that higher values indicate greater consumption of healthy and less consumption of unhealthy foods.

#### Secondary endpoints

Secondary outcomes were: (1) Cancer-related fatigue (Brief Fatigue Inventory, 9 items, BFI-9 [50, 51]), (2) Cancer-related emotional impact (Intrusion scale of the Impact of Event Scale-Revised, 7 items, IES-R [52]), (3) Depression (Patient Health Questionnaire, 9 items, PHQ-9 [53]), (4) Anxiety (Generalized Anxiety Disorder, 7 items, GAD-7 [54]), (5) Fear of progression (Fear of Progression questionnaire, 12 items, PA-F12 [55]), (6) Insomnia symptoms (Insomnia Severity Index, 7 items, ISI [56]), and (7) Subjective usefulness of the programme (single item, Net promoter score [57]), plus items to assess potential negative effects of the programme (23 items, Inventory for the Assessment of negative effects of psychotherapy—online version [German *Inventar zur Erfassung negativer Effekte von Psychotherapie*, *INEP-ON*] [58]. Further detail on all outcome measures and their psychometric properties is provided in the study protocol [34].

### Sample size calculation and analysis

As described in the study design paper [34], an a priori power analysis was performed with g*power (Version 3.1.9.2) [59] and showed that a sample size of 346 participants would be required to detect a small to medium effect (Cohen’s *d* = 0.35) with a power of 0.80 and an alpha level of 0.0167 (Bonferroni adjustment because of the three primary endpoints; 0.05/3). The anticipated effect size of Cohen’s *d* = 0.35 was deemed realistic based on previous reviews of psychosocial outcomes in cancer patients [60–62]. Therefore, we aimed to enrol 180 participants per group.

Missing data were replaced using multiple imputation (100 imputations, 50 iterations) based on sociodemographic data and available outcomes. Intention to treat (ITT) analyses were performed with data from all randomized participants. Results of per protocol (PP) analyses are presented in the S1 and S2 Tables and used data from all control group participants plus data from intervention group participants who had used the intervention for at least 60 minutes on at least four separate occasions. Analyses of Covariance (ANCOVA) were conducted for the primary outcomes to test for between-group differences at the 3-month time-point. Baseline values of the respective outcome were used as covariates in these analyses. For analyses involving the primary outcomes, a Bonferroni adjustment was used to address multiplicity and control for family-wise error rate (*p* < 0.05/3 = *p* < 0.017) [63]. For all other analyses, a p-level of 0.05 (two-tailed) was used. A sensitivity analysis was conducted for the primary outcomes using a conservative multiple imputation approach (reference-based multiple imputation using the jump to reference [J2R]) assumption rather than the missing at random (MAR) assumption. To examine potential dose-response relationships, correlational coefficients between usage and pre-post change in primary outcome variables were inspected. Statistical analyses were performed with R-Studio 1.3 (incl. mice package 3.11.0), BlueSky Statistics 7.0, and with STATA-SE Version 16.

## Results

### Description of trial participants

Participants were recruited between October 25, 2018 (first patient in) and April 6, 2020 (last patient out). Demographic characteristics are presented in Table 1. The mean age of the women participating in this study was 50 years (range = 30–70). Most participants had attained a relatively high educational level, with 42% holding a university degree. The majority (70%) of participants were working full-time (31%) or part-time (39%), and most were married (69%) or in a relationship (14%). A minority of the women in the sample (29%) were currently in psychotherapy. The prototypical participant was a 50-year-old, well educated, married woman who is not undergoing psychotherapy and had not experienced tumour recurrence in the preceding three months.

**Table 1:** Demographic characteristics (at baseline).

	IG	CG	Total sample
	n = 181	n = 182	n = 363
**Age in years, mean (SD)**	50.07 (8.51)	49.8 (7.98)	49.93 (8.24)
**Education (highest degree)**			
basic-level high school (Hauptschule)	2	7	9
medium-level high school (Mittlere Reife)	32	32	64
higher-level high school (Fachhochschulreife)	19	17	36
highest-level high school (Abitur)	14	14	28
vocational education (Berufsausbildung)	30	24	54
university degree	76	76	152
other educational qualification	8	12	20
**Employment status**			
employed (full-time)	53	61	114
employed (part-time)	76	64	140
unemployed	37	35	72
other	15	22	37
**Family status**			
married	127	122	249
married (separated)	6	7	13
single	9	14	23
partnership	25	27	52
divorced	11	10	21
widowed	3	2	5
**Recurrence (past 3 months)**			
none	165	164	329
1	12	17	29
>1	4	1	5
**Respiratory infection (past 3 months)**			
none	109	107	216
≥ 1	72	75	147
**Unplanned Physician Visits (past 3 months)**			
none	79	83	162
≥ 1	102	99	100

*Note*. IG: intervention group, CG: control group, SD: standard deviation.

### Intervention delivery and drop-out

**Fig 1:**
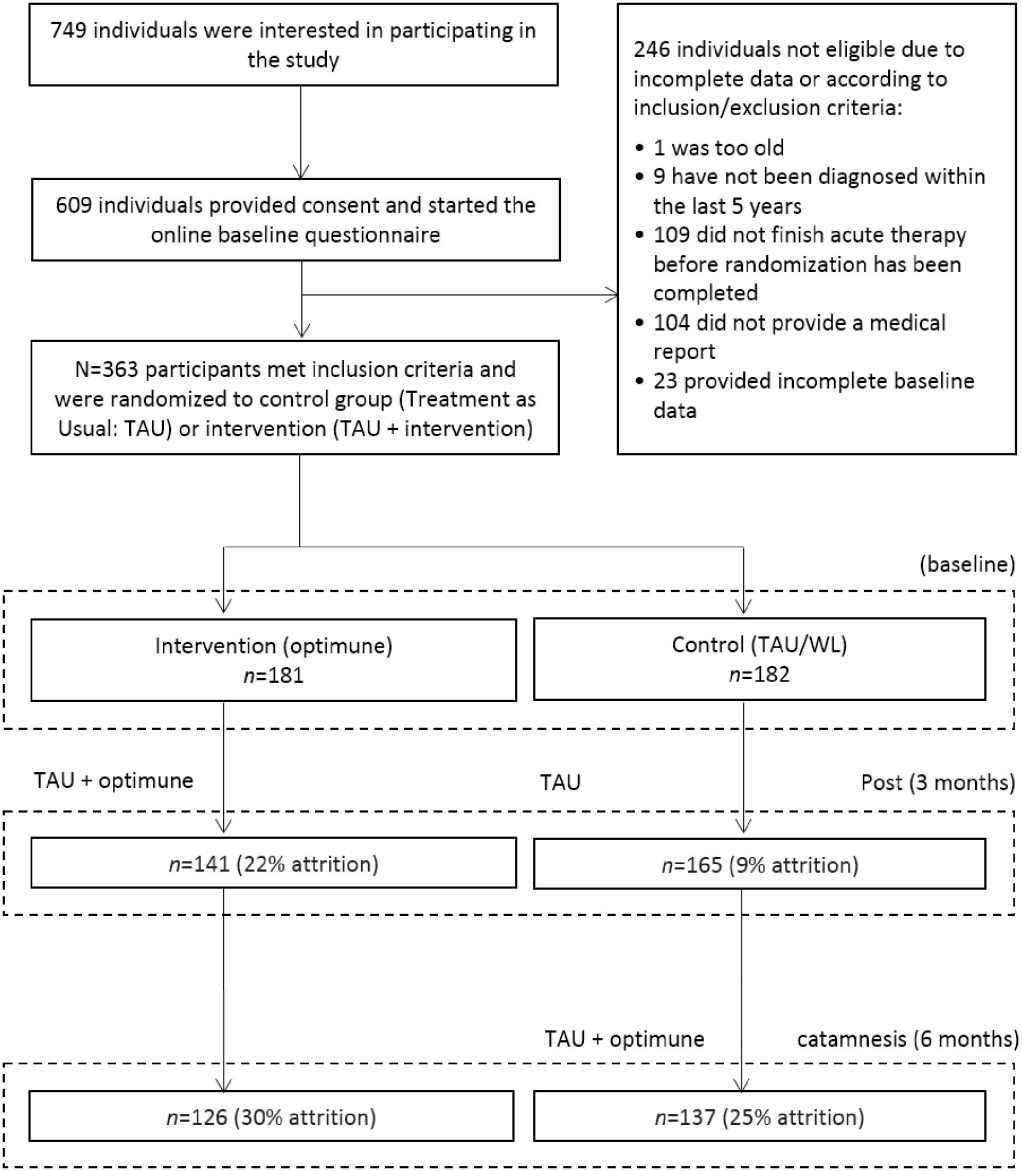
Study flowchart. Trial design and participant flow. TAU (Treatment as Usual), WL (waitlist).

In total, *N* = 363 participants were randomized to the IG (*n* = 181) or the CG (*n* = 182). The drop-out rate at 3 months (T1) in the total sample was 16% (22% in the IG and 9% in the CG). The study flowchart (Fig 1) provides further detail on reasons for dropout.

### Psychometric properties of the FQQ

Internal consistency of the total FQQ (Cronbach’s alpha = 0.81) as well as the two subscales healthy foods (alpha = 0.75) and unhealthy foods (alpha = 0.80) was acceptable to good. A moderate inverse correlation between the healthy and unhealthy foods subscales indicated that, as expected, participants who tend to eat healthy foods are somewhat less likely to also consume unhealthy foods, and vice versa (*r* = -0.32, *p* < 0.001). A principal components analysis (PCA) with varimax rotation, which is recommended for such exploratory analyses [64], suggested that four factors could be distinguished: (1) Vegetables and fatty fish (7 items), (2) Sweets (4 items), (3) Fast food (6 items), (4) Fruits and berries (3 items). When variables based on these factors were entered into a second-order PCA with varimax rotation, a clear two-factor solution emerged: (1) Healthy foods (vegetables and fatty fish; fruits and berries), and (2) unhealthy foods (sweets; fast food). The total (20 items) FQQ scale and both subscales (10 items each) were approximately normally distributed, with no problematic skew or kurtosis (values ranged from -0.44 to 0.76). Further details of these analyses are available from the authors. In summary, given the high levels of internal consistency, plausible inverse correlation between the subscales, interpretable factor structure, and normal distribution, the FQQ was deemed to be an acceptable outcome measure for the analyses reported below.

### Study outcomes

#### Primary outcomes

**Fig 2:**
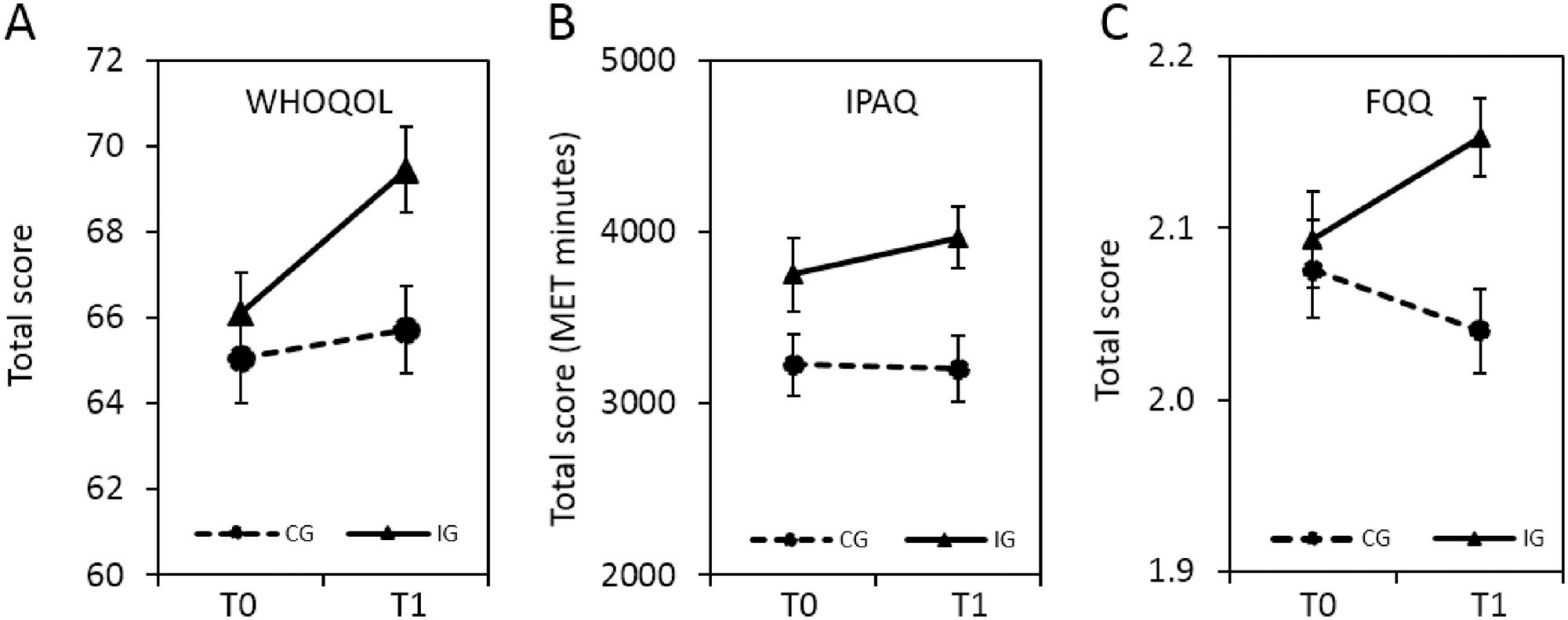
Primary endpoints. Effects of *Optimune* on quality of life, dietary habits and physical exercise habits. Mean total scores (error bars represent SEM) are shown for each outcome measure before the intervention (T0, baseline) and at the end of the intervention (T1, 3 months). Treatment had significant effects on (A) quality of life and (C) dietary habits, whereas there was no effect on (B) physical exercise. MET (metabolic equivalent task, in minutes per week), IG (intervention group, n = 181), CG (control group, n = 182).

ITT analyses showed significant effects on two of the three primary outcome scales at T1 (Table 2): Quality of life (95% CI: 0.07–0.48) and dietary habits (95% CI: 0.15–0.56). The third primary outcome, physical exercise, did not attain significance (95% CI: 0.10–0.51). Post-treatment between-group effect sizes ranged from 0.27 to 0.36 (Cohen’s *d*), corresponding to an NNT of 6.58 to 5.00 [65]. Fig 2 depicts the pre to post changes in primary outcome scales in both groups. Table 1 provides a summary of the subscale analyses of the primary outcome measures, revealing the relatively strongest effects for psychological quality of life (*d* = 0.42), healthy dietary habits (*d* = 0.36), and aerobic exercise (*d* = 0.32). Per-protocol (PP) analyses confirmed significant intervention effects on quality of life and dietary quality (see S1 Table).

**Table 2:** ITT analysis of primary endpoints.

		Pre (T0, Baseline)		Post (T1, 3 Months)		WG Effect Size	BG Effect Size
						Pre-Post	Post
		M	SD	M	SD	Cohen’s *d* (95% CI)	Cohen’s *d* (95% CI)
**Quality of Life total**	IG	66.11	14.22	69.44	13.63	0.24 (0.03–0.45)	0.27 (0.07–0.48)
	CG	65.05	12.86	65.70	13.56	0.05 (-0.16–0.25)	
Physical	IG	64.88	17.31	71.69	14.14	0.43 (0.22–0.64)	0.31 (0.10–0.52)
	CG	63.76	18.28	66.81	17.11	0.17 (-0.03–0.38)	
Psychological	IG	61.48	16.32	68.24	13.79	0.45 (0.24–0.66)	0.42 (0.21–0.63)
	CG	59.96	16.84	62.07	15.67	0.13 (-0.08–0.34)	
Social	IG	62.50	20.73	60.48	19.24	-0.10 (-0.31–0.11)	-0.04 (-0.25–0.16)
	CG	62.05	18.80	61.25	18.30	-0.04 (-0.25–0.16)	
Environment	IG	75.67	14.26	77.01	12.96	0.10 (-0.11–0.30)	0.22 (0.01–0.43)
	CG	74.84	13.04	74.17	12.99	-0.05 (-0.26–0.15)	
**Physical Activity total MET**	IG	3749	2867	3967	2456	0.08 (-0.12–0.29)	0.30 (0.10–0.51)
	CG	3223	2437	3198	2580	-0.01 (-0.22–0.20)	
anaerobic MET	IG	1047	1267	1049	919	0.00 (-0.20–0.21)	0.12 (-0.09–0.33)
	CG	1081	1321	931	1044	-0.13 (-0.33–0.08)	
aerobic MET	IG	997	1164	1314	1372	0.25 (0.04–0.46)	0.32 (0.11–0.53)
	CG	854	1119	900	1203	0.04 (-0.17–0.25)	
walk MET	IG	1545	1433	1577	1197	0.02 (-0.18–0.23)	0.23 (0.02–0.44)
	CG	1282	1411	1296	1240	0.01 (-0.19–0.22)	
sit MET	IG	2502	1166	2378	1041	0.11 (-0.09–0.32)	0.22 (0.01–0.43)
	CG	2613	1190	2621	1155	0.01 (-0.20–0.21)	
**Dietary Habits total**	IG	2.09	0.38	2.15	0.30	0.17 (-0.03–0.38)	0.36 (0.15–0.56)
	CG	2.08	0.38	2.04	0.33	-0.10 (-0.31–0.10)	
Healthy	IG	1.86	0.47	2.01	0.45	0.33 (0.12–0.54)	0.36 (0.15–0.56)
	CG	1.83	0.48	1.84	0.49	0.02 (-0.18–0.23)	
Unhealthy	IG	0.67	0.46	0.56	0.39	0.26 (0.05–0.47)	0.13 (0.08–0.33)
	CG	0.68	0.45	0.61	0.40	0.16 (-0.05–0.37)	

Note. Results of ITT analysis of primary endpoints. anaerobic (time spent with anaerobic, strenuous activity), aerobic (time spent with aerobic activity), walk (time spent walking), sit (time spent sitting), MET (metabolic equivalent task, in minutes per week), IG (intervention group), CG (control group), M (mean), SD (standard deviation), WG (within group), BG (between group), CI (confidence interval), Pre (time point of baseline, before start of intervention, T0), Post (time point of 3 months after start of intervention, T1).

#### Secondary outcomes

As summarized in Table 3, significant intervention effects were observed in the ITT analyses for four of the six secondary outcome scales: insomnia, cancer-related fatigue, general anxiety symptoms, and depressive symptoms. By contrast, intervention effects were not significant for cancer-related emotional stress and fear of tumour progression. PP analyses (see S2 Table) suggested a highly similar pattern of results, with significant effects on five of the six outcome scales (all but general anxiety).

**Table 3:** ITT analysis secondary endpoints.

		Pre (T0, Baseline)		Post (T1, 3 Months)		WG Effect Size	BG Effect Size
						Pre-Post	Post
		M	SD	M	SD	Cohen’s *d* (95% CI)	Cohen’s *d* (95% CI)
Insomnia	IG	12.08	6.04	10.22	5.72	0.32 (0.11–0.52)	0.28 (0.07–0.48)
	CG	12.08	5.72	11.78	5.59	0.05 (-0.15–0.26)	
Cancer-related Fatigue	IG	4.41	2.28	4.09	2.16	0.14 (-0.06–0.35)	0.23 (0.02–0.44)
	CG	4.71	2.24	4.61	2.28	0.04 (-0.16–0.25)	
Cancer-related Emotional Impact	IG	1.73	0.93	1.46	0.86	0.31 (0.10–0.51)	0.01 (-0.20–0.22)
	CG	1.59	1.01	1.45	0.94	0.14 (-0.06–0.35)	
Depression	IG	8.82	4.91	7.06	4.23	0.39 (0.18–0.59)	0.29 (0.09–0.50)
	CG	9.30	5.05	8.38	4.77	0.19 (-0.02–0.39)	
Anxiety	IG	7.97	4.80	6.83	4.02	0.26 (0.05–0.46)	0.09 (-0.12–0.29)
	CG	7.62	4.69	7.20	4.39	0.09 (-0.11–0.30)	
Fear of Tumour Progression	IG	36.23	9.32	34.07	9.09	0.24 (0.03–0.44)	0.10 (-0.11–0.30)
	CG	35.87	9.53	34.98	9.75	0.09 (-0.11–0.30)	

*Note*. Results of ITT analysis of secondary endpoints. IG (intervention group), CG (control group), M (mean), SD (standard deviation), WG (within group), BG (between group), CI (confidence interval), Pre (time point of baseline, before start of intervention, T0), Post (time point of 3 months after start of intervention, T1).

#### Stability of effects in the IG and improvements in the CG after 3 months

We inspected the stability of intervention effects by examining changes in the IG between 3 and 6 months, using data from the ITT analyses. We first considered data from IG participants only. Given that CG participants could access the programme after 3 months, their data were examined separately.

Among IG participants, the majority of treatment effects remained stable or improved further between 3 and 6 months (see S3 Table). Specifically, such stability was observed with regard to total QoL (also physical, psychological, and environment subscales), three of four physical exercise scales (anaerobic, walking, and sitting), the two dietary habits subscales, cancer-related emotional stress, and depressive symptoms. IG participants experienced further improvements between 3 and 6 months on several scales: the social quality of life subscale, total dietary habits, insomnia symptoms, general anxiety symptoms, and fear of cancer progression. However, participants in the IG also reported some reductions in physical activity (total scale and aerobic subscale) between 3 and 6 months.

Among CG participants, who received access to the intervention after 3 months, significant improvements were observed on most scales (see S3 Table): Total quality of life (also the physical, psychological, and environment subscales), total dietary habits (also both subscales), insomnia, cancer-related fatigue and emotional distress, depressive symptoms, general anxiety, and fear of cancer progression. The only exception was that physical activity levels remained stable among CG participants during this period. Overall, improvements were observed across a broad range of outcomes after control group participants were able to access the intervention.

#### Supplemental analyses suggesting immunological response

At baseline, the proportion of participants reporting at least one episode of a respiratory infection, common cold or influenza did not differ between the CG (41%) and IG (40%), χ^2^(1) = 0.77, *p* = 0.83. At 3-months, 38% of IG participants and 48% of CG participants reported at least one such episode, but this was not a statistically significant difference, χ^2^ (1) = 3.28, *p* = 0.08. At baseline, the proportion of participants reporting at least one unplanned physician visit did not differ between the IG (56%) and the CG (54%), χ^2^ (1) = 0.14, *p* = 0.75. However, at 3-months, 43% of IG participants versus 56% of CG participants reported at least one unplanned physician visit, which was a significant difference, χ^2^ (1) = 5.30, *p* = 0.02.

#### Adverse effects and user satisfaction

The evaluation of adverse events showed that an undesirable event related to the use of *Optimune* was reported by nine patients. In eight cases, this was increased distress (e.g., feeling upset because the content reminded the patient of the cancer diagnosis, feeling pressured to do exercises or change habits, feeling frustrated because response options did not match the personal situation). In one case, a patient reported an unintended reduction of body weight, which she attributed to eating less meat, sweets, juices and chips. None of the reported events were judged to be serious adverse events. Control group participants were not asked about adverse events. Participants were also asked to indicate whether they would recommend *Optimune* to a friend or colleague (“net promoter score” or NPR [57]). Responses were made on an 11-point scale ranging from 0 (not at all likely) to 10 (very likely). Of the 134 participants who responded to this item, 9% were not satisfied (0 to 3), 16.4% were somewhat satisfied (4 to 6), and 74.6% were satisfied to very satisfied (7 to 10). The mean of 7.71 (SD = 2.59) indicated that participants were rather likely to recommend the programme to others. Using the traditional approach to interpreting the NPR, a score of 24.6% was computed, which can be regarded as good.

#### Dose-response effects

Participants in the IG used *Optimune* for a mean number of 25.7 days (*SD* = 33.9). Frequency of intervention use did not correlate, however, with the amount of change in primary outcomes between baseline and post-treatment (3 months).

## Discussion

### Summary of main results

*Optimune*, the Internet intervention examined here, facilitated significant improvements in patients’ quality of life and in healthy dietary habits. More tentative support was found for improvements in physical exercise habits, but this effect did not reach statistical significance. Nevertheless, trial success had been defined a priori as reaching significance on at least one of the three primary outcomes; therefore, this study provided first evidence of the efficacy of *Optimune*, a novel Internet intervention designed to improve mental health and facilitate change in health behaviours relevant to immune function among breast-cancer survivors.

A more fine-grained analysis of the primary outcome subscales revealed several notable details. Firstly, with respect to QoL, intervention effects were strongest for the psychological QoL subscale, suggesting that patients experienced improvements in their mental health, self-esteem, and cognitive abilities (e.g., memory and concentration). A somewhat weaker but statistically significant effect was observed for the physical aspects of QoL, suggesting that the intervention improves QoL aspects such as activities of daily living, mobility, work capacity, and dependence on medical. By contrast, intervention effects on the social relations and environment QoL domains were smaller, indicating the facets such as personal relationships, financial resources, or the home environment are less likely to be affected by *Optimune*, as one might expect. In sum, these findings suggest that *Optimune* targets mental as well as physical health components of QoL, which is consistent with the intervention’s goal of reducing psychological stress, enhancing mental health, and bolstering physical health by improving immune functioning.

With regard to the second primary outcome, dietary habits, intervention effects were stronger for improvements in healthy dieting rather than reductions in unhealthy dieting. In concrete terms, patients in the IG reported eating more healthy foods, such as vegetables, fruit and fatty fish, but their tendency to also reduce consumption of unhealthy foods (e.g., high-sugar foods, highly industrially processed “junk food” or “fast food”) was less pronounced. The goal of *Optimune* was to improve dietary habits by consuming more foods that improve healthy immune system functioning while also reducing immune-compromising foods, so this goal seems to be at least partially achieved.

With regard to the outcome exercise habits, there was some evidence that patients in the IG tended to increase their level of aerobic activity. This is in line with the conceptual goals of *Optimune*, as regular aerobic exercise seems to reduce inflammatory markers, such as CRP, TNF-α, and Interleukin-6 [66], and reducing chronic inflammation may improve the prognosis of breast cancer survivors [10].

*Optimune* also yielded improvements in several secondary outcomes, including reductions in insomnia, cancer-related fatigue, depression, anxiety, and fear of tumour progression. Furthermore, few negative reactions to the intervention were observed, and no serious adverse events occurred. The majority of the users were satisfied with *Optimune* and likely to recommend it to others.

Finally, exploratory findings suggested that the intervention might affect immunological parameters. That is, fewer intervention than control group participants reported at least one unplanned physician visit during the 3 months of intervention usage, whereas there was no such difference in the preceding 3 months. Compared to control group participants, intervention group participants also reported fewer respiratory infections at three months but not at baseline; however, this was not a significant difference. Taken together, these findings suggest that the intervention might facilitate improvements in immune status, although studies with greater statistical power will be needed to examine such potential effects and, if confirmed, to study underlying biological mechanisms, including chronic inflammation.

### Significance of findings in light of previous research

Although it is difficult to define the exact threshold for a clinically relevant effect [67, 68], the majority of effects observed in this trial may be clinically relevant. For example, evidence suggests that effects of *d* = 0.24 can be regarded as clinically relevant with respect to psychosocial outcomes such as depression [69]. Applying this criterion, all of the intervention effects on primary outcomes and several effects on secondary outcomes can be deemed clinically relevant.

The effects observed in this trial are generally in line with effects observed for other behavioural interventions for cancer patients on psychosocial outcomes, such as distress and quality of life [60–62]. For example, one meta-analysis reported small to medium effects of various CBT-based interventions on distress and pain in breast cancer patients; however, after adjusting for differences in sample size and treatment format (individual versus group), effect sizes for larger and group CBT formats were small [60]. Another systematic review reported that mindfulness-based stress reduction interventions had an average effect of Cohen’s d = 0.29 on QoL, which is similar to the effect we observed [61].

Of note, it has been argued that the value of an intervention does not depend solely on the magnitude of its effect size but also on its capacity to reach large numbers of individuals, because interventions that are easily scalable could improve population health even if effect sizes are modest [27]. As an Internet intervention that does not require personal support and can be used on any computer or smartphone with a web browser, *Optimune* can be regarded as a prototype of a highly scalable intervention.

In line with several meta-analyses, this trial confirmed that CBT can be delivered effectively via an Internet intervention without the provision of personal support or guidance [30, 40, 70, 71]. Previous meta-analyses have shown that self-guided Internet interventions achieve average effects of *d* = 0.27, for example, when depression is targeted as the primary endpoint [71]. Personal guidance can enhance overall treatment effectiveness, but the incremental effects of support provision appear to be surprisingly small, and the qualification of the support providers might make little difference (e.g., highly qualified clinicians vs. less qualified technicians) [72–74]. Additionally, self-guided or minimally supported Internet interventions may be more cost-efficient and easier to implement, enhancing their potential to impact health on a population level [25, 62]. From a methodological perspective, it is also important to examine the effects of Internet interventions without the potential confounder of personal support by research staff, which may only be available when the intervention is implemented in routine care settings. In Germany, the Federal Institute for Drugs and Medical Devices, which evaluates whether digital health interventions are deemed reimbursable, also mandates trials to demonstrate that interventions are effective without the provision of services such as clinician guidance [75].

Breast cancer survivors often do not receive the psychosocial support they require, particularly once they have moved past the acute treatment phase [24], and many of them recognize the advantages and opportunities provided by Internet-based support, such as flexible remote access [76]. In line with this trial, several other studies have now demonstrated the effectiveness of Internet interventions for breast cancer survivors on outcomes such as quality of life, pain severity, fear of tumour progression, fatigue and insomnia [77–79]. Digital interventions also have the potential to improve exercise and dietary habits among breast cancer survivors, according to meta-analytic evidence [80]. However, although the potential of such interventions has been shown repeatedly, the methodological quality of many studies is poor (e.g., small pilot studies), and many such interventions are narrow in focus (e.g., targeting only insomnia) and may not be available in routine practice. The implementation of evidence-based digital interventions remains a formidable challenge and requires the consideration of multiple barriers on different levels (e.g., organizational and care structures, patient-related, provider-related, reimbursement) [81].

### Strengths and limitations

Advantages of this trial were that it was adequately powered to detect clinically relevant effects, that diagnoses at study entry were confirmed by clinicians, that a follow-up assessment was included to examine the stability of effects, that an array of well validated outcome measures was used, and that potential adverse effects were specifically examined. Several limitations must also be acknowledged. For example, this trial did not include an alternative specific active treatment comparator, although CAU control groups are generally favoured in pragmatic trials, which aim to examine the effects of behavioural interventions in realistic and heterogeneous routine care conditions [43]. Another limitation is that a novel questionnaire was used to assess dietary habits. Although an effort was made to ensure that the content of this questionnaire was aligned with current evidence [47–49] and with the conceptual content of *Optimune*, and initial psychometric characteristics are promising, further studies are needed to establish its construct validity. However, the success of *Optimune* in this trial did not depend on the result obtained with this novel questionnaire, as the effect on quality of life was significant. We also acknowledge the limitation that outcomes were measured exclusively by patient self-reports, whereas biological indicators of health or immune status could increase objectivity. Another limitation is that we collected little data on variables such as time since breast cancer diagnosis, stage of disease, and prior treatments as well as current therapy, which makes it difficult to compare this sample with others. Finally, we note that this sample was relatively highly educated and included individuals who were motivated and able to engage with an internet intervention. Patients from under-represented minorities, older adults, rural populations, and those with lower health and computer literacy may be more difficult to reach with interventions such as the one we examined. Therefore, the results may not be generalizable to the entire population of breast cancer survivors. However, we note that *Optimune* is intended to be used by patients who have access to the internet and who have the cognitive capacities to engage with it.

### Conclusion and future directions

In conclusion, this trial provided evidence for the safety and efficacy of *Optimune*, a new CBT-based, holistic Internet intervention for breast cancer patients. The intervention did not cause serious adverse effects and can therefore be regarded as safe, it was associated with few negative emotional reactions, and it led to statistically significant and clinically relevant improvements in quality of life and dietary habits. The intervention also led to improvements in several secondary outcomes, including insomnia, depression, anxiety, fear or tumour progression, and cancer-related emotional distress. Finally, compared to CG participants, IG participants reported fewer unplanned physician visits over 3 months, which suggests that it might facilitate improvements in general health and possibly immune status. We conclude that *Optimune* could be disseminated more broadly to breast cancer patients, many of whom do not routinely receive psychosocial support or CBT. It would be desirable to replicate these effects and to examine whether they are mediated by immunological processes, such as reductions in inflammation. Given its scalability, efficiency, flexibility and ease of implementation, we anticipate that *Optimune* has the potential to improve the quality of life and general health of breast cancer patients. Moreover, we anticipate that versions of *Optimune* could be developed in other languages and for other target populations, given that improvements in immune status, mental and physical health, as well as dietary and exercise habits are beneficial for a broad spectrum of the general population.

## Supporting information

S1 File Sensitivity analyses (reference-based multiple imputation). (DOCX)

S2 File Minimal anonymized data set. (XLS)

S1 Appendix Ethics committee application. (DOCX)

S1 Table PP analysis of primary endpoints. (DOCX)

S2 Table PP analysis of secondary endpoints. (DOCX)

S3 Table Follow-up results. Changes between 3 and 6 months (CG accessed intervention after 3 months).
(DOCX)

S1 Checklist (DOC)
