# Supplementary material for: Results of the Optimune trial: A randomized controlled trial evaluating a novel Internet intervention for breast cancer survivors
Source: PLoS One. 2021 May 7;16(5):e0251276. doi: 10.1371/journal.pone.0251276 (PMC8104369; doi:10.1371/journal.pone.0251276)
Supplement: S1 File — (DOCX) [file pone.0251276.s001.docx]

**S1 File.** **Sensitivity Analyses (reference-based multiple imputation)**

Sensitivity analyses were carried out for the primary endpoints quality of life (World Health Organization Quality of Life Questionnaire; WHOQOL-BREF), physical activity (International Physical Activity Questionnaire; IPAQ) and dietary habits (Food Quality Questionnaire; FQQ) at the pre-specified timepoint T1 (3-months).

A reference-based multiple imputation approach was used to impute the missing data points, as described in Carpenter et al. (2). The imputation was carried out using the cemimix program (3) in Stata version 16 (4).

Missing data points at the primary timepoint were imputed using the respective variable values ​​at baseline as well as sociodemographic and clinical variables (age, psychotherapy status, recurrence rate, respiratory infection rate, marital status, educational level, employment status). The imputation was stratified according to the intervention arm. A set of 100 imputations was performed.

As part of the sensitivity analysis, missing at random (MAR) results were compared with a reference-based imputation. This was based on the assumption that missing data from the intervention group followed a similar pattern to values ​​from the control group (Jump to Reference, J2R). In the J2R assumption, it is assumed that missing values ​​in the intervention group correspond to a lack of intervention effect and are thus replaced by values ​​from the control group.

Sensitivity Analysis of Primary Endpoints (T1, 3-months)

| Endpoint | Missing data assumption | optimune  M | control  M | Between Group Difference | | |
| --- | --- | --- | --- | --- | --- | --- |
|  |  |  |  | M  [95% CI] | Cohen’s *d*  [95% CI] | p-value |
| Quality of Life  (WHOQOL) | MAR | 69.57 | 65.73 | -3.85  [-4.13 to -3.56] | 0.28  [0.06 to 0.47] | 0.003 |
|  | J2R | 68.66 | 65.73 | -2.95  [-3.24 to -2.66] | 0.21  [-0.01 to 0.40] | 0.048 |
| Physical Activity  (IPAQ) | MAR | 3959 | 3233 | -727  [-783 to -670] | 0.26  [0.03 to 0.45] | 0.023 |
|  | J2R | 3847 | 3233 | -615  [-672 to -558] | 0.22  [0.00 to 0.41] | 0.054 |
| Dietary Habits  (FQQ) | MAR | 2.15 | 2.04 | -0.11  [-0.119 to -0.106] | 0.34  [0.12 to 0.54] | <0.001 |
|  | J2R | 2.13 | 2.04 | -0.08  [-0.095 to -0.081] | 0.26  [0.04 to 0.45] | 0.002 |

M = mean value, CI = Confidence Interval, MAR = Missing at Random, J2R = Jump to Reference

Assuming that all missing data points from drop-out participants in the intervention group have no treatment effect and therefore correspond to the imputation estimates of the control group (J2R assumption), the primary endpoint quality of life reaches p = 0.048 (Cohen’s d = 0.21), which is not below the Bonferroni-corrected significance level of *p* < 0.0167. However, even under the J2R assumption, the primary endpoint quality of life was below a *p*-value of 0.05. It can be concluded that with two conservative measures (Bonferroni correction and J2R assumption) the formally required level of significance was not achieved. However, it must be taken into account that the probability of a type II error (incorrect confirmation of the null hypothesis with a correct alternative hypothesis) is increased by these two measures. Since the intervention effect on improvement in quality of life is significant with the MAR assumption (with Bonferroni correction) and J2R (without Bonferroni correction), this can be interpreted as evidence for the effectiveness of optimune (with the reservations described above).

The sensitivity analyses for the endpoints physical activity and dietary habits confirm the effects under the original MAR-assumption. The intervention effect on improvement of dietary habits remains significant even with the J2R assumption. Since significance for one of the three endpoints (after Bonferroni correction) was required for the overall confirmation of efficacy, it can be concluded that the study demonstrated the efficacy of the intervention.

^1^ Holtdirk F, Mehnert A, Weiss M, Mayer J, Meyer B, Bröde P, Claus M, Watzl C (2020) Results of the Optimune trial: a randomized controlled trial evaluating a novel Internet intervention for breast cancer survivors. (PlosOne, in Revision)

^2^ Carpenter, JR., et al. (2013). Analysis of longitudinal trials with protocol deviation: a framework for relevant, accessible assumptions, and inference via multiple imputation. Journal of biopharmaceutical statistics, 23(6), 1352-1371.

^3^ Leurent, B., et al. (2020). Reference‐based multiple imputation for missing data sensitivity analyses in trial‐based cost‐effectiveness analysis. Health economics, 29(2), 171-184.

^4^ StataCorp. 2019. Stata Statistical Software: Release 16. College Station, TX: StataCorp LLC.
