# Supplementary material for: Results of the Optimune trial: A randomized controlled trial evaluating a novel Internet intervention for breast cancer survivors
Source: PLoS One. 2021 May 7;16(5):e0251276. doi: 10.1371/journal.pone.0251276 (PMC8104369; doi:10.1371/journal.pone.0251276)
Supplement: S1 Appendix — (DOCX) [file pone.0251276.s003.docx]

**S1 APPENDIX: Ethics committee application**

1. English translation: page 1-11
2. German original: page 12-23

**Application for ethical approval:**

**Evaluation of a Psychological Intervention as Additional Care for Women with Breast Cancer.**

Applicant: Prof. Dr. rer. nat. Carsten Watzl, IfADo (PI)

Co-operation partner: Dr. Björn Meyer,

Franziska Scheibe, M.A. (trial manager)

GAIA AG

Hans-Henny-Jahnn-Weg 53

22085 Hamburg

Prof. Dr. Anja Mehnert

Abteilung für Medizinische Psychologie und

Medizinische Soziologie & Sektion Psychosoziale Onkologie

Universitätsklinikum Leipzig AöR

Philipp-Rosenthal-Straße 55

04103 Leipzig

**1. Introduction**

***Background and Therapy***

In Germany 70.000 women are diagnosed with breast cancer every year. German guidelines (Kreienberg et al., 2013) recommend several acute therapies to be applied within the first months, i.e. surgery, radiotherapy and aggressive combination-chemotherapy. Patients who completed the initial intensive therapy and those who can be considered as cancer-free should be monitored and, if needed, treated with specific subsequent therapies, such as anti-hormonal therapy, aromatase-inhibitors, or bisphosphonate. During acute initial treatment phase patients often receive brief psychological support to be able to cope with the situation. However, psychological therapy is not part of care-as-usual (CAU) for patients with breast cancer. Even patients who are repeatedly tested negative for further symptoms of cancer during years of routine monitoring often exhibit other health-related problems. It was reported that 40% of breast cancer survivors suffer from depression and 27% develop anxiety disorder. It has also been shown that such symptoms can persist for more than five years after initial diagnosis (Maass et al., 2015; Von Ah and Kang, 2008). Moreover, an average of 27% of breast cancer survivors suffer from clinically significant fatigue symptoms after finishing acute therapy (Abrahams et al., 2016; Bower et al., 2006).

***Depression, Fatigue und Inflammation***

There is growing evidence for the association of depression and increased pro-inflammatory markers (Dowlati et al., 2010; Leonard, 2017). A causal link between both, however, still remains inconclusive. There are studies showing serotonin reuptake inhibitors might attenuate IL-6 and TNF-α levels. Other potent antidepressant drugs, however, do not appear to reduce pro-inflammatory cytokines (Hannestad et al., 2011).

Interestingly, several studies have found improved depressive symptoms after administration of anti-inflammatory drugs (NSAIDs and cytokine inhibitors) (Kohler et al., 2014; Raison et al., 2013). Symptoms of fatigue have also been associated with increased pro-inflammatory cytokine level (Louati and Berenbaum, 2015; Staud, 2015). Breast cancer survivors often show both increased inflammatory markers and symptoms of fatigue (Bower et al., 2002; Collado-Hidalgo et al., 2006; Pierce et al., 2009). A putative connection among fatigue and depression and inflammatory markers has also been discussed (Bower et al., 2002; Collado-Hidalgo et al., 2006; Dowlati et al., 2010). A prospective cohort study showed that elevated inflammatory markers predicted lower survival rates among patients with breast cancer (Pierce et al., 2009). Furthermore, depression was found to be associated with higher mortality among patients with breast cancer (Hjerl et al., 2003).

***Psychological Interventions***

Psychological intervention techniques, including mindfulness-based stress reduction (MBSR), cognitive behavioral therapy (CBT) and supportive-expressive psychotherapy (SE) have been shown to be effective in the treatment of depressive symptoms while also improving profiles of pro-inflammatory markers (IL-6, TNF- α) related to different psychiatric as well as somatic medical conditions with co-morbid depression (Del Grande da Silva et al., 2016; Moreira et al., 2015; Walsh et al., 2016). A recent meta-analysis found Mindfulness Meditation to mitigate inflammatory markers CRP and NF-kB, although no effects on other cytokines were reported (Black and Slavich, 2016). A randomized controlled trial (RCT) with breast cancer patients showed reduced fatigue and inflammation after Yoga practice. This trial further indicated a trend towards fewer depressive symptoms among participants who received the intervention (Kiecolt-Glaser et al., 2014). Several studies showed improved quality of life as well as reduced inflammatory markers in patients who underwent psychological interventions like CBT mindfulness-based treatments. (Bower et al., 2015; Carlson et al., 2003; McGregor and Antoni, 2009).

***‚eHealth’-Interventions***

Computer-based, individually tailored interventions could facilitate access to psychological interventions. GAIA has developed several computer-based interventions, which are tailored to individual patients suffering from, among others, depressive symptoms, multiple sclerosis (MS) with comorbid depression, or anxiety disorders. These interventions have been demonstrated to be effective in a number of RCTs. An online intervention developed by GAIA (deprexis) has been tested by several independent trials to be an effective treatment for people with depression (Beevers et al., 2017; Berger et al., 2011, in press; Fischer et al., 2015; Klein et al., 2016; Meyer et al., 2009; Meyer et al., 2015; Moritz et al., 2012; Schroder et al., 2014; Zwerenz et al., 2017). A meta-analysis on deprexis revealed an effect size of *d* = 0.54 (Twomey et al., 2017). This is a relevant effect size considering that an effect size of *d* = 0.45 has been reported for CBT, the “gold standard” psychotherapy for depression (Barth et al., 2013). Another meta-analysis further showed larger effects of deprexis compared to other electronic interventions for depression (Karyotaki et al., 2017). Two other e-health interventions, a program for anxiety disorders and another one focusing on fatigue in MS were both developed by GAIA and have been shown in RCTs to be effective treatments (Berger et al., 2017)(Pöttgen et al., 2018).

More e-health interventions developed by GAIA are being investigated in RCTs (Meyer et al., 2017; Zill et al., 2016); addressing depression, anxiety, alcohol-use disorder and epilepsy. Based on similar therapeutic principles as the previously developed web-based psychological interventions for conditions like depression, anxiety and fatigue, a new intervention program has been developed. Breast cancer survivors exhibit a set of psychological symptoms that considerably overlap with the aforementioned conditions. Associations of such a pattern of symptoms and inflammatory markers has been investigated and discussed, highlighting a promising approach for a new version of an internet intervention designed and developed to treat patients who have completed an acute breast cancer treatment (for details, see attachment 1). Additional to CBT addressing depression, anxiety and fatigue, the intervention includes a set of elements and modules tackling relevant health behaviors (dietary and physical exercise habits) and stress management (mindfulness), which might have effects on relevant immune and inflammatory markers. All aspects of psychological and behavioral methods comprised in the intervention are in line with current treatment guidelines “2016 AGO Richtlinien” (Arbeitsgemeinschaft Gynäkologische Onkologie e.V.) (Kuemmel and Schmidt, 2016). Guidelines include mindfulness-based stress reduction (MBSR), Yoga, physical exercise, smoking cessation, healthy diet (more healthy fatty acids, normal BMI, low alcohol intake). The techniques incorporated in the developed intervention (CBT, stress reduction, psycho-education) are also in line with the guidelines of the “Arbeitsgemeinschaft Psychoonkologie” (PSO; working group on psycho-oncology) of the “Deutsche Krebsgesellschaft (DKG, German Cancer Society)” (2014).

***Hypothesis***

The present study will test the hypothesis that the internet-based holistic psychological intervention “optimune” has an effect on lifestyle parameters and psychometric parameters among patients with breast cancer who have completed acute treatment and are currently free of recurrence. The immune system is essential in the fight against cancer. Supporting immune function through lifestyle adjustments is therefore a promising strategy for helping cancer patients. Thus, administering this psychological intervention in breast cancer survivors could not only reduce depressive symptoms and fatigue, but also lower inflammatory parameters and at the same time strengthen the immune system to help it fight tumor cells.

**2. Protocol and Design**

***Trial Design***

The study is conceived as a prospective, randomized, single-blind study with a waiting list control group design in which the patients are not blinded to conditions, but the persons carrying out the study are blinded. For details on implementation and timing, see schedule in Appendix 2.

The intervention program used is considered to be an "information website" for regulatory purposes and is therefore not a medical product. The study is therefore not a pivotal efficacy trial for regulatory purposes, but is of a purely exploratory nature.

***Recruitment of Participants***

Participants will be recruited using print media and web-based announcements, followed by a web-based questionnaire. Before the participants give their consent, they receive information about the goals and procedures used in the study and are informed that they can withdraw from the study at any time without negative consequences.

***Inclusion Criteria***

Participants of the trial can be women who:

- are between 30 and 70 years of age
- were diagnosed with breast cancer less than 5 years ago
- completed acute breast cancer treatment at least 1 month ago. This applies to operations, chemotherapy or radiation therapy - the date on which the last of these therapies was completed applies here. (A prophylactic anti-hormone treatment with e.g. tamoxifen, aromatase inhibitors or bisphosphonates is possible).
- are able to speak German
- declare consent to participate in the study
- provide a discharge letter from an oncology treatment centre (proving diagnosis and type of therapy)

***Protocol***

For details regarding procedures of the trial and time table see attachment 2. Prior to any participation in the trial participants have to submit a discharge letter (copy or scan via e-mail) and declare consent.

***Intervention***

Participants will be randomized and allocated to study groups after initial screening (questionnaires, see attachment). Participants in the intervention group receive access to the intervention program via a voucher sent by e-mail (360 days; details described in attachment 1) additionally to care-as-usual. The participants are supposed to follow the procedural instruction of the intervention program. Participants allocated to the waitlist control group will be informed about the procedure to receive delayed access to the intervention. After data acquisition via psychometric tests 90 days post inclusion, intervention group participants will keep access to the program for longitudinal analysis. At the same time, participants of the control group receive 360 days access to the program. Each participant who did not start using the program until 7 days after receiving access or will be contacted by a trial manager via e-mail or phone. Additionally, participants who are not using the program for two weeks will be encouraged via e-mail to actively engage with the intervention.

***Target Sample Size***

The estimation of the sample size to detect effects of the three primary endpoints between groups was based on standard assumptions: type-I error (α = 0.05), type-II error (β = 0.2), power (1-β = 0.8). The primary endpoints are continuous variables and will be tested for statistical differences using a student-t-test. An optional transformation may be applied to correct for non-normality of the sample. For a mean effect size of f = 0.25 a total sample size of 180 participants (90 in each group) will be needed (Cohen, 1992). Considering an attrition rate of 50% along the trial a final total number of participants N = 360 are planned to be recruited and included in the trial.

**3. Methods**

***Psychometric Tests***

At defined timepoints (T1: 90 and T2: 180 days after allocation) participants will be asked to complete an online assessment of data (psychometric endpoints and life-style parameters) via a set of questionnaires reported independently.

***Adverse Events***

During usage of the program each participant is supposed to complete a short survey about adverse events of online interventions which coincides with T1 and T2.

**Endpoints**

***Primary Endpoints***

The three primary endpoints will be assessed using online questionnaires.

- WHO QOL BREF
- IPAQ
- FQQ

Analysis of covariance of data at T1 (T2 as longitudinal) will consider the corresponding T0 (longitudinal: T1 respectively) variable as covariate.

***Secondary Endpoints***

Assessment of secondary endpoints will cover 6 arears: sleep, fatigue, cancer-related emotional stress, depression, anxiety, fear of tumor progression.

- ISI
- BFI
- IES-R (Intrusions-Subskala)
- PHQ-9
- GAD-7
- PA-F12

For further details regarding the questionnaires see attachment 3.

***Other Endpoints***

- recurrence of breast cancer
- incidence of infections (common cold, flu)
- incidence of unplanned physician visits
- height and weight
- concomitant psychotherapy

***Longitudinal Follow-Up***

The above-mentioned primary and secondary endpoints will be assessed 90 days (T1) and 180 days (T2) after allocation to trial group or individual start of the study respectively.

***Statistical Methods***

Linear models will be applied to use baseline values as covariates of the statistical analysis (Littell et al., 2006), that includes correlational and MANOVA-based techniques. Additionally, generalized linear models will be used for the analysis of frequency or incidence and binary or categorical data (Stokes et al., 2012) from secondary and other endpoints. Those methods are available in standard statistic software (SAS V9.2). The above-mentioned analyses will be applied to the entire sample following the principle of intention-to-treat (ITT) analysis (Gupta, 2011). Additionally, a per-protocol (PP) analysis will be performed to prevent bias by withdrawal, noncompliance and drop-in (controls act like intervention group). Definition of PP-analysis follows the pre-defined criterion of suggested usage of the program. Based on the scientific rationale and mode of action leading to the hypothesis of the trial, data will be tested with PP-analysis for effects according to intended usage (sufficient “exposure to treatment”, availability of data, no violation of the protocol) (EMEA, 1998). Participants who fulfill the following post-randomization-exclusion criteria will be excluded from PP-analyses:

- no valid data points for primary endpoints (intervention group at T1, control group at T2)
- recurrence of cancer and readmission of therapy (chemotherapy, radiotherapy, surgery)
- start of a psychotherapy during the course of the trial (face-to-face, group, web-based, minimum of two sessions per month)
- not used the intervention program at all
- not used for a minimum of 60 minutes or 4 sessions; Meyer et al., 2015.

Incidences of exclusion will be monitored and checked for systematic patterns (systematic difference between groups) (EMEA, 1998).

**4. Information und Consent**

Participant information about the trial as well as a form for written consent are attached.

**5. Confidentiality**

***Randomization***

Participants who fulfill the inclusion criteria will be allocated randomly to one of two equally sized study groups (intervention group versus waiting-list control group). Information about the assignment will be send out via e-mail representing the individual start date of the trial. For that purpose, three lists will be generated. List #1 (group disclosure) contains information about assignment of group A versus B to intervention versus control group. This list will be created by the trial manager and is supposed to be disclosed first with completion of data analysis after T2. List #2 (randomization sequence) will be used to randomly allocate IDs of participants to either one of group A or B using a block size of 4. This list will be generated by statistics software SAS version 9.2 to subsequently send out the above-mentioned e-mails. Only after completion of data acquisition at T2 the list will be disclosed to allow for blinded personnel involved in acquisition and analysis of the data set. List #3 assigns names of participants to randomized IDs ordered by date of admission to the trial. This list will be generated by the trial manager and is intended to enable the trial management to contact participants with reminder e-mails and monitor program usage. Besides, list #3 will exclusively be accessible for the trial manager; and never be disclosed to other personnel. Hence, trial manager is non-blind and able to work as administrator of the trial, yet not involved in data acquisition or analysis. Other personnel will work merely with participant IDs, not having access to personal data. The three lists will be AES-encoded and stored following data security policies.

***Blinding***

The proposed study is designed as a simple-blind trial with blinded personnel for data acquisition and analysis. Due to the procedural design of the trial participants can not be blinded to their assignment to a group. Technical support and support regarding content can be reached using a hotline which will be provided by personnel without involvement in handling data.

***Data Security***

Personal data (names of participants and contact information) will only be disclosed to the trial manager. Separation of access to information will be guaranteed by systematic lists for randomization. Additionally, personnel will be trained and reminded to keep confidentiality. For the presentation of data as talks, seminars and as publication no personal data or attributes will be used. Electronic storage of trial data will be carried out under pseudonyms; without retraceability. All data will be stored in encrypted file directories on protected servers.

**6. Legal Affairs**

There is no insurance for participants. Participants will be informed alongside the form for written consent about non-availability of insurance and usage of the software on one’s own responsibility; excluding liability.

**7. Communication of Results**

Results of data analysis will be published in a peer-reviewed journal.

**8. Referenzen**

(2014). Psychoonkologische Diagnostik, Beratung und Behandlung von erwachsenen Krebspatienten, Langversion 1.1. In AWMF-Registernummer: 032/051OL; http://leitlinienprogramm-onkologie.de/Leitlinien.7.0.html (Arbeitsgemeinschaft für Psychoonkologie (PSO) der Deutschen Krebsgesellschaft (DKG)).

Abrahams, H.J., Gielissen, M.F., Schmits, I.C., Verhagen, C.A., Rovers, M.M., and Knoop, H. (2016). Risk factors, prevalence, and course of severe fatigue after breast cancer treatment: a meta-analysis involving 12 327 breast cancer survivors. Ann Oncol *27*, 965-974.

Barth, J., Munder, T., Gerger, H., Nuesch, E., Trelle, S., Znoj, H., Juni, P., and Cuijpers, P. (2013). Comparative efficacy of seven psychotherapeutic interventions for patients with depression: a network meta-analysis. PLoS Med *10*, e1001454.

Beevers, C.G., Pearson, R., Hoffman, J.S., Foulser, A.A., Shumake, J., and Meyer, B. (2017). Effectiveness of an internet intervention (Deprexis) for depression in a united states adult sample: A parallel-group pragmatic randomized controlled trial. J Consult Clin Psychol *85*, 367-380.

Berger, T., Hammerli, K., Gubser, N., Andersson, G., and Caspar, F. (2011). Internet-based treatment of depression: a randomized controlled trial comparing guided with unguided self-help. Cogn Behav Ther *40*, 251-266.

Berger, T., Urech, A., Krieger, T., Stolz, T., Schulz, A., Vincent, A., Moser, C.T., Moritz, S., and Meyer, B. (2017). Effects of a transdiagnostic unguided Internet intervention ('velibra') for anxiety disorders in primary care: results of a randomized controlled trial. Psychol Med *47*, 67-80.

Berger, T., Krieger, T., Sude, K., Meyer, B., & Maercker, A. (in press). Evaluating an e-mental health program ("deprexis") as adjunctive treatment tool in psychotherapy for depression: Results of a pragmatic randomized controlled trial. Jn Affective Disorders. doi:10.1016/j.jad.2017.11.021

Black, D.S., and Slavich, G.M. (2016). Mindfulness meditation and the immune system: a systematic review of randomized controlled trials. Ann N Y Acad Sci *1373*, 13-24.

Bower, J.E., Crosswell, A.D., Stanton, A.L., Crespi, C.M., Winston, D., Arevalo, J., Ma, J., Cole, S.W., and Ganz, P.A. (2015). Mindfulness meditation for younger breast cancer survivors: a randomized controlled trial. Cancer *121*, 1231-1240.

Bower, J.E., Ganz, P.A., Aziz, N., and Fahey, J.L. (2002). Fatigue and proinflammatory cytokine activity in breast cancer survivors. Psychosom Med *64*, 604-611.

Bower, J.E., Ganz, P.A., Desmond, K.A., Bernaards, C., Rowland, J.H., Meyerowitz, B.E., and Belin, T.R. (2006). Fatigue in long-term breast carcinoma survivors: a longitudinal investigation. Cancer *106*, 751-758.

Carlson, L.E., Speca, M., Patel, K.D., and Goodey, E. (2003). Mindfulness-based stress reduction in relation to quality of life, mood, symptoms of stress, and immune parameters in breast and prostate cancer outpatients. Psychosom Med *65*, 571-581.

Claus, M., Dychus, N., Ebel, M., Damaschke, J., Maydych, V., Wolf, O.T., Kleinsorge, T., and Watzl, C. (2016). Measuring the immune system: a comprehensive approach for the analysis of immune functions in humans. Arch Toxicol *90*, 2481-2495.

Cohen, J. (1992). A power primer. Psychol Bull *112*, 155-159.

Collado-Hidalgo, A., Bower, J.E., Ganz, P.A., Cole, S.W., and Irwin, M.R. (2006). Inflammatory biomarkers for persistent fatigue in breast cancer survivors. Clin Cancer Res *12*, 2759-2766.

Committee for Human Medicinal Products (CHMP) (2017). Draft guideline on multiplicity issues in clinical trials (London, UK: European Medicines Agency).

Del Grande da Silva, G., Wiener, C.D., Barbosa, L.P., Goncalves Araujo, J.M., Molina, M.L., San Martin, P., Oses, J.P., Jansen, K., Dias de Mattos Souza, L., and Azevedo da Silva, R. (2016). Pro-inflammatory cytokines and psychotherapy in depression: Results from a randomized clinical trial. J Psychiatr Res *75*, 57-64.

Dowlati, Y., Herrmann, N., Swardfager, W., Liu, H., Sham, L., Reim, E.K., and Lanctot, K.L. (2010). A meta-analysis of cytokines in major depression. Biol Psychiatry *67*, 446-457.

EMEA (1998). ICH E9 Statistical principles for clinical trials (London, UK: European Medicines Agency).

Fischer, A., Schroder, J., Vettorazzi, E., Wolf, O.T., Pottgen, J., Lau, S., Heesen, C., Moritz, S., and Gold, S.M. (2015). An online programme to reduce depression in patients with multiple sclerosis: a randomised controlled trial. Lancet Psychiatry *2*, 217-223.

Gupta, S.K. (2011). Intention-to-treat concept: A review. Perspect Clin Res *2*, 109-112.

Hannestad, J., DellaGioia, N., and Bloch, M. (2011). The effect of antidepressant medication treatment on serum levels of inflammatory cytokines: a meta-analysis. Neuropsychopharmacology *36*, 2452-2459.

Hjerl, K., Andersen, E.W., Keiding, N., Mouridsen, H.T., Mortensen, P.B., and Jorgensen, T. (2003). Depression as a prognostic factor for breast cancer mortality. Psychosomatics *44*, 24-30.

Karyotaki, E., Riper, H., Twisk, J., Hoogendoorn, A., Kleiboer, A., Mira, A., Mackinnon, A., Meyer, B., Botella, C., Littlewood, E.*, et al.* (2017). Efficacy of Self-guided Internet-Based Cognitive Behavioral Therapy in the Treatment of Depressive Symptoms: A Meta-analysis of Individual Participant Data. JAMA Psychiatry *74*, 351-359.

Kiecolt-Glaser, J.K., Bennett, J.M., Andridge, R., Peng, J., Shapiro, C.L., Malarkey, W.B., Emery, C.F., Layman, R., Mrozek, E.E., and Glaser, R. (2014). Yoga's impact on inflammation, mood, and fatigue in breast cancer survivors: a randomized controlled trial. J Clin Oncol *32*, 1040-1049.

Klein, J.P., Berger, T., Schroder, J., Spath, C., Meyer, B., Caspar, F., Lutz, W., Arndt, A., Greiner, W., Grafe, V.*, et al.* (2016). Effects of a Psychological Internet Intervention in the Treatment of Mild to Moderate Depressive Symptoms: Results of the EVIDENT Study, a Randomized Controlled Trial. Psychother Psychosom *85*, 218-228.

Klein, J.P., Berger, T., Schroder, J., Spath, C., Meyer, B., Caspar, F., Lutz, W., Greiner, W., Hautzinger, M., Rose, M.*, et al.* (2013). The EVIDENT-trial: protocol and rationale of a multicenter randomized controlled trial testing the effectiveness of an online-based psychological intervention. BMC Psychiatry *13*, 239.

Kohler, O., Benros, M.E., Nordentoft, M., Farkouh, M.E., Iyengar, R.L., Mors, O., and Krogh, J. (2014). Effect of anti-inflammatory treatment on depression, depressive symptoms, and adverse effects: a systematic review and meta-analysis of randomized clinical trials. JAMA Psychiatry *71*, 1381-1391.

Kreienberg, R., Albert, U., Follmann, M., Kopp, I., Kühn, T., and Wöckel, A. (2013). Interdisziplinäre S3-Leitlinie für die Diagnostik, Therapie und Nachsorge des Mammakarzinoms. Senologie-Zeitschrift für Mammadiagnostik und-therapie *10*, 164-192.

Kuemmel, S., and Schmidt, M. (2016). AGO Guidelines Breast Version 2016.1D: Komplementäre Therapien Hormontherapie „Survivorship“ (Rezidiv-Prävention). https://wwwago-onlinede/fileadmin/downloads/leitlinien/mamma/Maerz2016/de/2016D%2024_Komplementaere%20Therapie.pdf [Stand: 18052017].

Ladwig, I., Rief, W., and Nestoriuc, Y. (2014). Welche Risiken und Nebenwirkungen hat Psychotherapie? - Entwicklung des Inventars zur Erfassung Negativer Effekte von Psychotherapie (INEP). Verhaltenstherapie *24*, 252-263.

Leonard, B.E. (2017). Inflammation and depression: a causal or coincidental link to the pathophysiology? Acta Neuropsychiatr, 1-16.

Littell, R.C., Milliken, G.A., Stroup, W.W., Wolfinger, R.D., and Schabenberger, O. (2006). SAS® System for Mixed Models, Second Edition (Cary, NC: SAS Institute Inc.).

Louati, K., and Berenbaum, F. (2015). Fatigue in chronic inflammation - a link to pain pathways. Arthritis Res Ther *17*, 254.

Maass, S.W., Roorda, C., Berendsen, A.J., Verhaak, P.F., and de Bock, G.H. (2015). The prevalence of long-term symptoms of depression and anxiety after breast cancer treatment: A systematic review. Maturitas *82*, 100-108.

McGregor, B.A., and Antoni, M.H. (2009). Psychological intervention and health outcomes among women treated for breast cancer: a review of stress pathways and biological mediators. Brain Behav Immun *23*, 159-166.

Meyer, B., Berger, T., Caspar, F., Beevers, C.G., Andersson, G., and Weiss, M. (2009). Effectiveness of a novel integrative online treatment for depression (Deprexis): randomized controlled trial. J Med Internet Res *11*, e15.

Meyer, B., Bierbrodt, J., Schröder, J., Berger, T., Beevers, C.G., Weiss, M., Jacob, G., Späth, C., Andersson, G., Lutz, W.*, et al.* (2015). Effects of an Internet intervention (Deprexis) on severe depression symptoms: Randomized controlled trial. Internet Interventions *2*, 48-59.

Meyer, B., Weiss, M., Holtkamp, M., Arnold, S., Brückner, K., Schröder, J., . . . Nestoriuc, Y. (2017). Protocol for the ENCODE trial: evaluating a novel online depression intervention for persons with epilepsy. BMC Psychiatry *17(1),* 55.

Moreira, F.P., Cardoso Tde, A., Mondin, T.C., Souza, L.D., Silva, R., Jansen, K., Oses, J.P., and Wiener, C.D. (2015). The effect of proinflammatory cytokines in Cognitive Behavioral Therapy. J Neuroimmunol *285*, 143-146.

Moritz, S., Schilling, L., Hauschildt, M., Schroder, J., and Treszl, A. (2012). A randomized controlled trial of internet-based therapy in depression. Behav Res Ther *50*, 513-521.

Pierce, B.L., Ballard-Barbash, R., Bernstein, L., Baumgartner, R.N., Neuhouser, M.L., Wener, M.H., Baumgartner, K.B., Gilliland, F.D., Sorensen, B.E., McTiernan, A.*, et al.* (2009). Elevated biomarkers of inflammation are associated with reduced survival among breast cancer patients. J Clin Oncol *27*, 3437-3444.

Porter, K.E. (2016). Statistical Power in Evaluations that Investigate Effects on Multiple Outcomes: A Guide for Researchers (New York, USA: MDRC).

Raison, C.L., Rutherford, R.E., Woolwine, B.J., Shuo, C., Schettler, P., Drake, D.F., Haroon, E., and Miller, A.H. (2013). A randomized controlled trial of the tumor necrosis factor antagonist infliximab for treatment-resistant depression: the role of baseline inflammatory biomarkers. JAMA Psychiatry *70*, 31-41.

Schroder, J., Bruckner, K., Fischer, A., Lindenau, M., Kother, U., Vettorazzi, E., and Moritz, S. (2014). Efficacy of a psychological online intervention for depression in people with epilepsy: a randomized controlled trial. Epilepsia *55*, 2069-2076.

Sheehan, D.V., Lecrubier, Y., Sheehan, K.H., Amorim, P., Janavs, J., Weiller, E., Hergueta, T., Baker, R., and Dunbar, G.C. (1998). The Mini-International Neuropsychiatric Interview (M.I.N.I.): the development and validation of a structured diagnostic psychiatric interview for DSM-IV and ICD-10. J Clin Psychiatry *59 Suppl 20*, 22-33;quiz 34-57.

Staud, R. (2015). Cytokine and immune system abnormalities in fibromyalgia and other central sensitivity syndromes. Curr Rheumatol Rev *11*, 109-115.

Stokes, M., Davis, C., and Koch, G. (2012). Categorical Data Analysis Using SAS®, Third Edition (Cary, NC: SAS Institute).

Twomey C, O'Reilly G, Meyer B. Effectiveness of an individually-tailored computerised CBT programme (Deprexis) for depression: A meta-analysis. Psychiatry Res. 2017 Oct;256:371-377. doi: 10.1016/j.psychres.2017.06.081. Epub 2017 Jun 27. PMID: 28686935.

Von Ah, D., and Kang, D.H. (2008). Correlates of mood disturbance in women with breast cancer: patterns over time. J Adv Nurs *61*, 676-689.

Walsh, E., Eisenlohr-Moul, T., and Baer, R. (2016). Brief mindfulness training reduces salivary IL-6 and TNF-alpha in young women with depressive symptomatology. J Consult Clin Psychol *84*, 887-897.

Zill, J. M., Meyer, B., Topp, J., Daubmann, A., Härter, M., & Dirmaier, J. (2016). Vorvida: study protocol of a randomized controlled trial testing the effectiveness of Internet-based self-help program for the reduction of alcohol consumption for adults. BMC Psychiatry, *16*, 19.

Zwerenz, R., Becker, J., Knickenberg, R. J., Siepmann, M., Hagen, K., & Beutel, M. E. (2017). Online Self-Help as an Add-On to Inpatient Psychotherapy: Efficacy of a New Blended Treatment Approach. Psychotherapy and Psychosomatics *86,* 341-350.

**Ethikantrag zur Studie:**

**Wissenschaftliche Überprüfung eines psychologischen Interventionsprogramms als zusätzliches Hilfsmittel für Frauen mit Brustkrebs**

Antragssteller: Prof. Dr. rer. nat. Carsten Watzl, IfADo (Studienleitung)

Kooperationspartner: Dr. Björn Meyer,

Franziska Scheibe, M.A. (Studienmanager)

GAIA AG

Hans-Henny-Jahnn-Weg 53

22085 Hamburg

Prof. Dr. Anja Mehnert

Abteilung für Medizinische Psychologie und

Medizinische Soziologie & Sektion Psychosoziale Onkologie

Universitätsklinikum Leipzig AöR

Philipp-Rosenthal-Straße 55

04103 Leipzig

**1. Fragestellung der Studie**

***Hintergrund zur Erkrankung und Therapie***

In Deutschland werden jährlich ca. 70.000 Frauen mit Brustkrebs diagnostiziert. Die deutschen Richtlinien (Kreienberg et al., 2013) empfehlen mehrere akute Therapien, welche innerhalb der ersten Monate durchgeführt werden sollen, wie z. B. Operationen, Strahlentherapie und aggressive Kombinations-Chemotherapie. Sobald diese Behandlungen abgeschlossen sind und die Patientinnen als krebsfrei gelten, folgt auf eine Phase der intensiven Behandlung, eine Phase mit lediglich standardisierten Kontrolluntersuchungen kombiniert mit einigen wenigen spezifischen Therapien (z. B. anti-hormonelle Therapie, Behandlungen mit Aromatase-Inhibitoren oder Bisphosphonat). Während viele Patientinnen in der Phase der akuten Behandlung kurzfristige psychologische Unterstützung erhalten, um mit der Situation besser fertig zu werden, gehört eine psychologische Betreuung nicht zur Standardbehandlung in den darauffolgenden Jahren. Obwohl Brustkrebspatientinnen während der Phase der Kontrolluntersuchungen frei von erneuten Krebssymptomen sind, haben sie oft andere Gesundheitsprobleme. Im Durchschnitt leiden 40 % der Brustkrebsüberlebenden an Depressionen und 27 % an Angstzuständen. Diese Symptome dauern normalerweise länger als 5 Jahre nach der Erstdiagnose an (Maass et al., 2015; Von Ah and Kang, 2008). Im Durchschnitt leiden 27% der Brustkrebsüberlebenden nach der akuten Behandlung an klinisch signifikanten Erschöpfungszuständen (Abrahams et al., 2016; Bower et al., 2006).

***Depression, Erschöpfung und Entzündungsparameter***

Es gibt viele Hinweise darauf, dass Depressionen mit erhöhter Entzündungsaktivität einhergehen (Dowlati et al., 2010; Leonard, 2017). Der kausale Zusammenhang ist jedoch weniger eindeutig. Während es Belege dafür gibt, dass Serotonin Wiederaufnahme-Hemmer die IL-6 und TNF-α Spiegel senken können, scheinen andere Antidepressiva zwar die depressiven Symptome zu verringern, nicht aber den Zytokinspiegel zu senken (Hannestad et al., 2011). Interessanterweise haben verschiedene Studien eine Besserung der Depression und von depressiven Symptomen nach der Verabreichung von entzündungshemmenden Wirkstoffen (sowohl NSAIDs, als auch Zytokininhibitoren) zeigen können (Kohler et al., 2014; Raison et al., 2013). Ebenso wurden Erschöpfungszustände mit einem erhöhten pro-inflammatorischen Zytokinspiegel in Zusammenhang gebracht (Louati and Berenbaum, 2015; Staud, 2015). Es überrascht daher nicht, dass in Brustkrebsüberlebenden höhere Spiegel von Entzündungsparametern beobachtet wurden (Bower et al., 2002; Collado-Hidalgo et al., 2006; Pierce et al., 2009), welche höchstwahrscheinlich im Zusammenhang mit den Depressionen und Erschöpfungszuständen dieser Patientinnen stehen (Bower et al., 2002; Collado-Hidalgo et al., 2006; Dowlati et al., 2010). Darüber hinaus stellte eine prospektive Kohortenstudie fest, dass erhöhte Entzündungsbiomarker mit verringerten Überlebensraten von Brustkrebspatientinnen korrelieren (Pierce et al., 2009) und dass Depressionen mit einem höheren relativen Mortalitätsrisiko dieser Patientinnen einhergehen (Hjerl et al., 2003).

***Verhaltensinterventionen***

Verschiedene psychologische Interventionen, einschließlich achtsamkeitsbasierte Stressreduktion (MBSR), kognitive Verhaltenstherapie (CBT) und supportiv-expressive psychodynamische Psychotherapie haben gezeigt, dass sie nicht nur die Depressionssymptome verbessern können, sondern auch Auswirkungen auf Entzündungsmarker haben, wie z.B. IL-6 oder TNF-α bei verschiedenen Krankheiten, z.B. psychiatrische Erkrankungen oder somatische Krankheiten mit komorbider Depression (Del Grande da Silva et al., 2016; Moreira et al., 2015; Walsh et al., 2016). Eine kürzlich durchgeführte Meta-Analyse zeigte, dass Achtsamkeitsmeditation einen Einfluss auf einige Entzündungsmarker wie CRP oder NF-kB hat, jedoch keine Auswirkungen auf eine Vielzahl anderer Zytokine zeigt (Black and Slavich, 2016). Unlängst konnte bei einer randomisierten kontrollierten Studie aufgezeigt werden, dass spezifisch bei Brustkrebspatientinnen Yoga-Praxis dazu führte, Erschöpfungszustände und Entzündungen deutlich zu reduzieren. Weiterhin zeigte sich ein Trend zur Reduktion von depressiven Symptomen (Kiecolt-Glaser et al., 2014). Verschiedene Studien haben zudem gezeigt, dass psychologische Interventionen, wie z. B. CBT und achtsamkeitsbasierte Behandlungen, bei Krebspatienten sowohl zu einer Verbesserung der Lebensqualität, als auch zu einer Reduktion von Entzündungsparametern führen können (Bower et al., 2015; Carlson et al., 2003; McGregor and Antoni, 2009).

***‚eHealth’-Interventionen***

Computerbasierte, individuell zugeschnittene Interventionen bieten die Möglichkeit, psychologische Interventionen leichter zugänglich zu machen. GAIA hat computerbasierte Interventionen entwickelt, die auf Patienten zugeschnitten sind, die an depressiven Symptomen, mit Multipler Sklerose assoziierten Depressionen oder Angststörungen leiden. Diese Interventionen haben ihre Wirksamkeit in verschiedenen randomisierten klinischen Studien (RCTs) gezeigt. Im Besonderen zeigten zehn RCTs die Wirkung von „Deprexis“, einer GAIA Intervention für Depression (Beevers et al., 2017; Berger et al., 2011, in press; Fischer et al., 2015; Klein et al., 2016; Meyer et al., 2009; Meyer et al., 2015; Moritz et al., 2012; Schroder et al., 2014; Zwerenz et al., 2017). Eine kürzlich abgeschlossenen Meta-Analyse zeigte eine Effektstärke von d = 0,54 für „Deprexis“ (Twomey et al., 2017). Dies ist vergleichbar ist mit dem Wert von d = 0,45 für den Gold-Standard der psychotherapeutischen Behandlung von Depressionen, nämlich der persönlichen kognitiven Verhaltenstherapie (CBT) (Barth et al., 2013). Eine Meta-Analyse auf Patientenebene zeigte zudem, dass Deprexis größere Effekte hatte als andere auf Depression fokussierende eInterventionen (Karyotaki et al., 2017). Eine auf Angststörungen ausgerichtete eHealth Intervention von GAIA zeigte außerdem ihre Wirkung in einem RCT (Berger et al., 2017). Auch konnte eine auf Erschöpfungszustände fokussierende GAIA Intervention ihre Wirkung bei Patientinnen mit Multipler Sklerose aufzeigen (Pöttgen et al., under review). Mehrere ähnliche eHealth Interventionen von GAIA werden aktuell in RCTs untersucht (Meyer et al., 2017; Zill et al., 2016); diese zielen auf Themen wie Depressionen, Ängstlichkeit, Reduktion von schädlichem Alkoholkonsum, sowie gesunder Ernährung und körperliche Aktivität bei Epilepsie-Patienten ab.

Aufbauend auf der beschriebenen Wirkung von GAIAs web-basierten psychologischen Interventionen bei Krankheitszuständen wie Depression, Angstzuständen und Erschöpfungszuständen, welche üblicherweise bei Brustkrebsüberlebenden auftreten, und bei denen angenommen wird, dass sie im kausalen Zusammenhang mit Entzündungen stehen, wurde unlängst eine neue Version der Internet-Intervention entwickelt (Einzelheiten, siehe Anhang 1). Neben etablierten CBT Techniken, welche sich auf Depression, Angstzustände und Erschöpfungszustände konzentrieren, beinhaltet diese Intervention zusätzliche Elemente, welche Auswirkungen auf Immun- und Entzündungsparameter gezeigt haben. Diese umfassen z. B. Schlaf- und Stressmanagement (z. B. Techniken basierend auf Achtsamkeit) sowie Lifestyle Optimierung (Beratung hinsichtlich Ernährung und körperlicher Betätigung). Diese zusätzlichen Aspekte, welche im Programm angesprochen werden, stehen im Einklang mit den empfohlenen alternativen Therapien der 2016 AGO Richtlinien (Arbeitsgemeinschaft Gynäkologische Onkologie e.V.) (Kuemmel and Schmidt, 2016): auf Achtsamkeit basierte Stressreduktion, Yoga, körperliche Betätigung, Rauchverzicht, Einhaltung eines gesunden, nährstoffreichen Ernährungsplans gemäß anerkannter Empfehlungen, wie z.B. Reduzierung des Fettkonsums, Ansteuern eines normalen BMI, Vermeidung von Alkoholkonsum >6g/Tag. Techniken, welche im Programm verwendet werden wie z. B. CBT, Entspannungstechniken und psycho-edukative Interventionen, (z. B. Stressmanagement, Krankheitsinformationen, Schulungen zum Thema gesunder Lifestyle), werden auch von den Richtlinien der Arbeitsgemeinschaft Psychoonkologie (PSO) der Deutschen Krebsgesellschaft (DKG) (2014) empfohlen.

***Hypothese***

Die aktuelle Studie wird die Hypothese überprüfen, dass eine Internet-basierte ganzheitliche psychologische Intervention, eine Wirkung auf Lebensstil-Parameter und psychometrische Parameter bei Brustkrebspatientinnen hat, welche sich in der Phase nach der aktiven Bekämpfung befinden und nicht vom Wiederauftreten der Krankheit betroffen sind. Das Immunsystem ist unentbehrlich beim Kampf gegen den Krebs. Die Immunfunktionen durch eine Anpassung des Lebensstils zu unterstützen ist daher eine vielversprechende Strategie, um Krebspatientinnen zu helfen. Somit könnte eine psychologische Intervention bei Brustkrebsüberlebenden nicht nur die depressiven Symptome und Erschöpfungszustände verringern, sondern auch die Entzündungsparameter senken und gleichzeitig das Immunsystem stärken, um es bei seiner Bekämpfung von Tumorzellen zu unterstützen.

**2. Studienplanung und Studiendesign**

***Studiendesign***

Die Studie ist als prospektive, randomisierte, Einfach-Blindstudie mit einem Warte- Kontrollgruppendesign konzipiert, in der nicht die Patientinnen, sondern die Studiendurchführenden verblindet werden. Für Einzelheiten bezüglich der Durchführung und Zeitabläufe, siehe Zeitplan in Anhang 2.

Das verwendete Interventionsprogramm gilt regulatorisch als eine "Informations-Website" und ist daher auch kein Medizinprodukt. Die Studie wird dementsprechend nicht als Zulassungsstudie hierfür benötigt, sondern sie ist rein explorativer Natur.

***Vorauswahl von Teilnehmerinnen***

Die Teilnehmerinnen werden mit Hilfe von Printmedien und web-basierten Bekanntmachungen, gefolgt von einem web-basierten Fragebogen rekrutiert. Bevor die Teilnehmerinnen ihr Einverständnis geben, erhalten diese Informationen über die Ziele und Vorgehensweisen, welche in der Studie benutzt werden und werden darüber informiert, dass sie zu jedem Zeitpunkt von der Studie ohne negative Folgen zurücktreten können.

***Einschlusskriterien***

An der Studie können Frauen teilnehmen:

- die 30 -70 Jahre alt sind
- bei welchen vor weniger als 5 Jahren Brustkrebs diagnostiziert wurde
- welche vor mindestens 1 Monat die akute Behandlung für Brustkrebs abgeschlossen haben. Dies gilt für Operationen, Chemotherapie oder Bestrahlungstherapie – es gilt hier das Datum an welchem die letzte dieser Therapien abgeschlossen wurde. (Eine prophylaktische anti-Hormonbehandlung mit z. B. Tamoxifen, Aromatasehemmern oder Bisphosphonaten ist möglich).
- die der deutschen Sprache mächtig sind
- die ihr Einverständnis zur Studie geben
- die bereit sind, den Entlassungsbrief aus der Onkologie vorzulegen (um die Diagnose und die Therapien zu belegen)

***Vorgehensweisen bei der Studie***

Zu Einzelheiten der Maßnahmen dieser Studie und zu Zeitabläufen, siehe Zeitplan aufgeführt in Anhang 2.

Vor jeglichen Studienaktivitäten, müssen die Teilnehmerinnen der Einwilligungserklärung zustimmen, sowie den Entlassungsbrief aus der Onkologie vorlegen (als Kopie oder Scan per E-Mail, um die Diagnose und die Therapien zu überprüfen).

***Die Intervention***

Nach einer Eingangsbefragung (Fragebögen, siehe unten) werden die Teilnehmerinnen randomisiert. Die Teilnehmerinnen, die der Interventionsgruppe zugeteilt wurden, erhalten zusätzlich zu ihrer normalen Behandlung 360 Tage lang Zugang zu einem ganzheitlichen psychologischen Interventionsprogramm, welches im Anhang 1 detailliert beschrieben wird. Die Teilnehmerinnen sollten die Übungen wie im Programm vorgeschrieben durchführen. Die Teilnehmerinnen der Interventionsgruppe erhalten in einer Randomisierungs-E-Mail einen Zugangsvoucher, sowie Informationen für das weitere Vorgehen. Die E-Mails der Warte-Kontrollgruppe enthalten Informationen zum verzögerten Zugang zur Intervention. Nach Durchführung der psychometrischen Tests nach 90 Tagen, wird für die Interventionsgruppe der Zugang zum Programm für weitere 90 Tage in der nachfolgenden Längsschnittphase weitergehen. Gleichzeitig wird den Teilnehmerinnen der Warte-Kontrollgruppe für 360 Tage Zugang zu dem Programm gewährt. Die Teilnehmerinnen der Interventionsgruppe werden per E-Mail oder telefonisch vom Studienmanager kontaktiert werden, wenn sie 7 Tage nach Erhalt der Zugangsdaten das Programm noch nicht begonnen haben oder wenn sie sich im weiteren Verlauf der Studie für einen Zeitraum von 2 Wochen das Programm nicht benutzt haben, und werden dazu aufgefordert werden, das Programm aktiv zu nutzen.

***Schätzung der Stichprobengröße***

Die Schätzung der Stichprobengröße zur Untersuchung des Unterschieds in der Veränderung vom Ausgangswert der drei primären Endpunkte (PEP, s.u.) zwischen den beiden unabhängigen Behandlungsgruppen, basierte auf Standardannahmen für die Typ-I-Fehlerrate (α=0.05) und Typ-II-Fehlerrate (β=0.2) d.h. Teststärke (Power)=1-β=0.8. Da die PEPs auf einer stetigen Skala gemessen werden, wird der Student-t-Test angewandt, um Differenzen auf statistische Signifikanz zu prüfen, optional nachdem die Variablen transformiert wurden, um eine bessere Annäherung an die Normalverteilung zu erlangen, wie es für PEP ratsam sein könnte.

Um eine mittlere Effektstärke, d.h. eine f von 0,25 als statistisch signifikant zu erkennen, was ein übliches Kriterium in ähnlichen explorativen Studien darstellt, wird unter diesen Voraussetzungen eine Stichprobengröße von schätzungsweise 180 Patientinnen (d. h. 90 in jeder Gruppe) benötigt (Cohen, 1992). Unter Berücksichtigung einer Abbruchquote von 50 % werden wir eine Gesamtanzahl von 360 Patientinnen für unsere Studie rekrutieren.

**3. Erhebungsinstrumente**

***Psychometrische Tests***

Zu vorab festgelegten Zeitpunkten (bei Studienbeginn (Randomisierung), am 90. Tag nach Beginn der Randomisierung sowie am 180. Tag nach Beginn der Randomisierung), werden die Teilnehmerinnen aufgefordert, eine web-basierte Selbsteinschätzung für die oben erwähnten psychometrischen Endpunkte und Lebensstil-Parameter durchzuführen. Selbsteinschätzungen für die Zeitpunkte werden vom Studienmanager per E-Mail oder telefonisch terminiert.

***Überwachung von Nebenwirkungen***

Während die Teilnehmerinnen Zugang zu dem Programm haben, müssen diese alle 90 Tage ein Freitextfeld ausfüllen, um potentielle negative Effekte von onlinebasierten psychotherapeutischen Interventionen einzuschätzen.

**Endpunkte**

***Primäre Endpunkte (PEP)***

Drei Lebensstilparameter, die anhand der Online-Fragebögen ermittelt werden, werden als co-primäre Endpunkte verwendet. Unterschiede in der Veränderung zum Ausgangswert zwischen den beiden Studiengruppen nach 90 Tagen ab der Randomisierung werden für die drei Bereiche Lebensqualität, Bewegung und Ernährung untersucht, bewertet durch:

- WHO QOL BREF
- IPAQ
- FQQ

Die Ausgangswerte jedes einzelnen Endpunkts werden in der Analyse als eine Kovariate verwendet.

***Sekundäre psychometrische Endpunkte***

Unterschiede in der Veränderung zum Ausgangswert zwischen den beiden Studiengruppen nach 90 Tagen ab der Randomisierung werden 6 Bereiche beinhalten (Schlaf, Erschöpfung, krebsbedingter emotionaler Stress/krebsbezogene intrusive Gedanken, Depressivität, Ängstlichkeit, und Angst vor Tumor-Progression), bewertet durch:

- ISI,
- BFI,
- IES-R (Intrusions-Subskala)
- PHQ-9
- GAD-7
- PA-F12.

Für Einzelheiten der verwendeten Fragebogen, siehe Anhang 3.

***Andere Endpunkte***

Unterschiede zwischen den beiden Studiengruppen nach 90 bzw. 180 Tagen ab der Randomisierung, werden untersucht auf:

- Wiederauftreten von Brustkrebs (Lokalrezidiv oder Fernmetastase)
- Häufigkeit von normalen Erkältungen oder Grippen
- Häufigkeit von ungeplanten Arztbesuchen
- Größe und Gewicht
- Psychotherapie

***Längsschnitt-Nachuntersuchung nach Abschluss des randomisierten Hauptstudienzeitraums (180 Tage nach Beginn der Randomisierung)***

Die oben erwähnten primären und sekundären Endpunkte werden 90 und 180 Tage nach Beginn der Randomisierung erneut überprüft.

***Statistische Methoden für individuelle Endpunkte (Fokus auf primäre Endpunkte)***Die Einbeziehung von Ausgangswerten (baseline) jedes Endpunkts als Kovariate in die statistische Analyse erfolgt durch die Applikation linearer Modelle (Littell et al., 2006), welche Korrelations- und MANOVA basierte Techniken umfassen. Gleichermaßen werden sogenannte generalisierte lineare Modelle (Stokes et al., 2012) für die Analyse von Häufigkeits- und Zähldaten, Ereignisraten oder binären Daten verwendet werden, welche sich durch die Analyse der sekundären und anderer Endpunkte ergeben. Alle diese Methoden sind in statischen Software-Programmen wie z. B. SAS® V9.2 verfügbar. Diese Analysen werden an der gesamten Stichprobe, gemäß dem Intention-to-Treat-Prinzip (ITT-Prinzip) durchgeführt, welches kurz als „einmal randomisiert, immer analysiert“ beschrieben werden kann (Gupta, 2011). Um jedoch potentielle Verzerrungen, welche durch Dinge wie Rücktritt, Nichtbefolgung, Abweichungen vom Protokoll (einschließlich drop-in, d. h. Kontrollpersonen verhalten sich wie die Interventions-Personen) u. ä. hervorgerufen werden, zu überprüfen, werden die Ergebnisse mit denen einer Per-Protokoll (PP)- Analyse verglichen. Diese basiert auf einer reduzierten Stichprobe, die definiert wird als „Datensätze, welche durch die Teilmenge von Teilnehmerinnen, die das Protokoll hinreichend befolgten, generiert werden“. Dadurch soll sichergestellt werden, dass diese Daten die Wirkung der Behandlung widerspiegeln, gemäß des zugrundeliegenden wissenschaftlichen Modells. Befolgung beinhaltet Gesichtspunkte wie ‚exposure to treatment’, Vorhandensein von Messwerten und das Fehlen von groben Protokollverstößen (EMEA, 1998).

Teilnehmerinnen, auf welche die folgenden Post-Randomisierungs-Ausschlusskriterien zutreffen werden von der PP-Analyse ausgeschlossen:

- Teilnehmerinnen (sowohl der Interventions- als auch der Kontrollgruppe) ohne gültige Daten für die jeweiligen primären Endpunkte, welche am 90. Tag (Interventionsgruppe) bzw. 180. Tag ermittelt werden (Kontrollgruppe).
- Teilnehmerinnen (sowohl der Interventions- als auch der Kontrollgruppe) bei denen der Krebs erneut aufgetreten ist und bei denen eine Behandlung mit Chemotherapie, Strahlentherapie oder eine Operation vor dem 180. Tag durchgeführt wird.
- Teilnehmerinnen (sowohl der Interventions- als auch der Kontrollgruppe) welche vor dem 180. Tag eine von der Studie unabhängige Psychotherapie begonnen haben (regelmäßige Psychotherapie, entweder 1:1, Gruppentherapie oder web-basierte Interaktion; mindestens zwei Sitzungen pro Monat).
- Teilnehmerinnen der Interventionsgruppe, welche das Interventionsprogramm noch gar nicht benutzt haben oder die es nicht im Rahmen der vordefinierten Mindestgrenze genutzt haben (mindestens 60 Minuten lang und mindestens 4 Sitzungen, Meyer et al., 2015).

Die Probleme, welche zum Ausschluss von Teilnehmerinnen führen, werden protokolliert und auf Muster, d. h. auf systematische Differenzen in ihrem Auftreten zwischen der Kontroll- und Interventionsgruppe, analysiert (EMEA, 1998).

**4. Probandeninformation und Einverständniserklärung**

Die Probandeninformation sowie die Einverständniserklärung sind dem Antrag beigefügt.

**5. Vertrauliche Behandlung der Daten**

***Randomisierung***

Die Teilnehmerinnen, welche die Einschlusskriterien erfüllen, werden willkürlich 2 gleichgroßen Studiengruppen zugeteilt (Interventionsgruppe gegenüber Warte-Kontrollgruppe). Die Gruppenzuteilung wird mit Hilfe von E-Mails erfolgen, die den Studienteilnehmerinnen bei Studienbeginn geschickt werden. Es werden drei Listen generiert werden: Liste 1 enthält die Informationen, welche Gruppe (Gruppe A oder Gruppe B) der Interventionsgruppe und welche der Warte-Kontrollgruppe zugeteilt wird. Diese Liste wird vom Studienmanager erzeugt und wird erst dann offengelegt, wenn die statistische Datenanalyse nach dem randomisierten Vergleichszeitraums von 180 Tagen abgeschlossen ist. Liste 2 (Randomisierungs-Sequenz), teilt mit einer Blockgröße von 4 willkürlich einer der beiden Studiengruppen (A oder B) Teilnehmer IDs zu. Diese Liste wird unter Verwendung der Statistiksoftware SAS® Version 9.2 generiert werden, und wird vom Studienmanager dafür verwendet werden, die oben erwähnten Emails zu erstellen. Diese Liste wird erst am Ende des randomisierten Vergleichszeitraums von 180 Tagen offengelegt werden, um die Verblindung der an Datenerhebung und –Auswertung beteiligten Mitarbeiter zu gewährleisten. Liste 3 ordnet in der Reihenfolge der Studienteilname die Namen der Teilnehmerinnen den randomisierten Teilnehmer IDs zu wird ebenfalls vom Studienmanager generiert. Diese Liste wird dafür verwendet werden, die Teilnehmerinnen für das Ausfüllen der Online-Fragebögen zu kontaktieren sowie die Programm-Nutzung zu monitoren und ggf. Erinnerungen zur aktiven Nutzung zu versenden. Diese Liste wird nur dem Studienmanager bekannt sein und wird zu keiner Zeit offengelegt. Somit ist der Studienmanager entblindet und hat alle nötigen Informationen zur administrativen Abwicklung der Studie. Der Studienmanager wird aber zu keinem Zeitpunkt in die Datenerhebung oder Auswertung involviert sein. Alle anderen Mitarbeiter der Studie (Mitarbeiter der Datenerfassung und der Datenauswertung) werden lediglich mit der Teilnehmer ID arbeiten ohne Zugang zu personenbezogenen Daten zu haben. Die drei Listen werden AES-verschlüsselt und datenschutzkonform beim Studienmanager aufbewahrt.

***Verblindung***

Bei dieser Studie handelt es sich um eine Einfach-Blindstudie mit Verblindung der Datenerheber und –Auswerter. Jedoch lässt es sich nicht vermeiden, dass sich die Teilnehmerinnen darüber bewusst sind, welcher Behandlungsgruppe sie angehören. Technische, sowie inhaltliche Unterstützung, welche das Interventionsprogramm betreffen, wird durch eine zentrale Hotline durch einen Mitarbeiter, welcher nicht in die Bewertung der oben beschriebenen Endpunkte involviert ist, gewährt.

***Datensicherheit***

Die Namen der Teilnehmerinnen und deren Kontaktinformationen werden nur dem Studienmanager bekannt sein. Diese Trennung von Informationen wird durch die drei im „Randomisierungs“ Abschnitt beschriebenen Listen garantiert. Zusätzlich dazu werden die Mitarbeiter geschult und daran erinnert, dass sie alle Informationen vertraulich behandeln müssen. Bei der Präsentation der Daten in Vorträgen, Seminaren und Veröffentlichungen, werden keine Namen oder identifizierende Merkmale erwähnt werden. Jegliche Informationen werden als vertrauliches Material behandelt und werden nur dem Forschungs- und klinischen Personal zur Verfügung stehen. Die elektronische Speicherung von Forschungsdaten erfolgt pseudonymisiert, d. h. nur mit der Teilnehmer ID versehen ohne Möglichkeit, die erhobenen Daten einer Person zuzuordnen. Alle Aufzeichnungen werden in verschlüsselten Ordnern und auf sicheren Servern aufbewahrt werden.

**6. Rechtliche Beziehungen**

Es besteht keine Probandenversicherung. Die Teilnehmerinnen werden im Aufklärungsbogen darüber informiert, dass für die Studie keine Probandenversicherung besteht und sie das Interventionsprogram auf eigene Verantwortung nutzen.

**7. Kommunikation der Ergebnisse**

Die Ergebnisse der Datenanalyse sollen Gegenstand wissenschaftlicher Publikationen sein. Ein Rückschluss auf ein einzelnes Individuum ist hierbei nicht möglich.

**8. Referenzen**

(2014). Psychoonkologische Diagnostik, Beratung und Behandlung von erwachsenen Krebspatienten, Langversion 1.1. In AWMF-Registernummer: 032/051OL; http://leitlinienprogramm-onkologie.de/Leitlinien.7.0.html (Arbeitsgemeinschaft für Psychoonkologie (PSO) der Deutschen Krebsgesellschaft (DKG)).

Abrahams, H.J., Gielissen, M.F., Schmits, I.C., Verhagen, C.A., Rovers, M.M., and Knoop, H. (2016). Risk factors, prevalence, and course of severe fatigue after breast cancer treatment: a meta-analysis involving 12 327 breast cancer survivors. Ann Oncol *27*, 965-974.

Barth, J., Munder, T., Gerger, H., Nuesch, E., Trelle, S., Znoj, H., Juni, P., and Cuijpers, P. (2013). Comparative efficacy of seven psychotherapeutic interventions for patients with depression: a network meta-analysis. PLoS Med *10*, e1001454.

Beevers, C.G., Pearson, R., Hoffman, J.S., Foulser, A.A., Shumake, J., and Meyer, B. (2017). Effectiveness of an internet intervention (Deprexis) for depression in a united states adult sample: A parallel-group pragmatic randomized controlled trial. J Consult Clin Psychol *85*, 367-380.

Berger, T., Hammerli, K., Gubser, N., Andersson, G., and Caspar, F. (2011). Internet-based treatment of depression: a randomized controlled trial comparing guided with unguided self-help. Cogn Behav Ther *40*, 251-266.

Berger, T., Urech, A., Krieger, T., Stolz, T., Schulz, A., Vincent, A., Moser, C.T., Moritz, S., and Meyer, B. (2017). Effects of a transdiagnostic unguided Internet intervention ('velibra') for anxiety disorders in primary care: results of a randomized controlled trial. Psychol Med *47*, 67-80.

Berger, T., Krieger, T., Sude, K., Meyer, B., & Maercker, A. (in press). Evaluating an e-mental health program ("deprexis") as adjunctive treatment tool in psychotherapy for depression: Results of a pragmatic randomized controlled trial. Jn Affective Disorders. doi:10.1016/j.jad.2017.11.021

Black, D.S., and Slavich, G.M. (2016). Mindfulness meditation and the immune system: a systematic review of randomized controlled trials. Ann N Y Acad Sci *1373*, 13-24.

Bower, J.E., Crosswell, A.D., Stanton, A.L., Crespi, C.M., Winston, D., Arevalo, J., Ma, J., Cole, S.W., and Ganz, P.A. (2015). Mindfulness meditation for younger breast cancer survivors: a randomized controlled trial. Cancer *121*, 1231-1240.

Bower, J.E., Ganz, P.A., Aziz, N., and Fahey, J.L. (2002). Fatigue and proinflammatory cytokine activity in breast cancer survivors. Psychosom Med *64*, 604-611.

Bower, J.E., Ganz, P.A., Desmond, K.A., Bernaards, C., Rowland, J.H., Meyerowitz, B.E., and Belin, T.R. (2006). Fatigue in long-term breast carcinoma survivors: a longitudinal investigation. Cancer *106*, 751-758.

Carlson, L.E., Speca, M., Patel, K.D., and Goodey, E. (2003). Mindfulness-based stress reduction in relation to quality of life, mood, symptoms of stress, and immune parameters in breast and prostate cancer outpatients. Psychosom Med *65*, 571-581.

Claus, M., Dychus, N., Ebel, M., Damaschke, J., Maydych, V., Wolf, O.T., Kleinsorge, T., and Watzl, C. (2016). Measuring the immune system: a comprehensive approach for the analysis of immune functions in humans. Arch Toxicol *90*, 2481-2495.

Cohen, J. (1992). A power primer. Psychol Bull *112*, 155-159.

Collado-Hidalgo, A., Bower, J.E., Ganz, P.A., Cole, S.W., and Irwin, M.R. (2006). Inflammatory biomarkers for persistent fatigue in breast cancer survivors. Clin Cancer Res *12*, 2759-2766.

Committee for Human Medicinal Products (CHMP) (2017). Draft guideline on multiplicity issues in clinical trials (London, UK: European Medicines Agency).

Del Grande da Silva, G., Wiener, C.D., Barbosa, L.P., Goncalves Araujo, J.M., Molina, M.L., San Martin, P., Oses, J.P., Jansen, K., Dias de Mattos Souza, L., and Azevedo da Silva, R. (2016). Pro-inflammatory cytokines and psychotherapy in depression: Results from a randomized clinical trial. J Psychiatr Res *75*, 57-64.

Dowlati, Y., Herrmann, N., Swardfager, W., Liu, H., Sham, L., Reim, E.K., and Lanctot, K.L. (2010). A meta-analysis of cytokines in major depression. Biol Psychiatry *67*, 446-457.

EMEA (1998). ICH E9 Statistical principles for clinical trials (London, UK: European Medicines Agency).

Fischer, A., Schroder, J., Vettorazzi, E., Wolf, O.T., Pottgen, J., Lau, S., Heesen, C., Moritz, S., and Gold, S.M. (2015). An online programme to reduce depression in patients with multiple sclerosis: a randomised controlled trial. Lancet Psychiatry *2*, 217-223.

Gupta, S.K. (2011). Intention-to-treat concept: A review. Perspect Clin Res *2*, 109-112.

Hannestad, J., DellaGioia, N., and Bloch, M. (2011). The effect of antidepressant medication treatment on serum levels of inflammatory cytokines: a meta-analysis. Neuropsychopharmacology *36*, 2452-2459.

Hjerl, K., Andersen, E.W., Keiding, N., Mouridsen, H.T., Mortensen, P.B., and Jorgensen, T. (2003). Depression as a prognostic factor for breast cancer mortality. Psychosomatics *44*, 24-30.

Karyotaki, E., Riper, H., Twisk, J., Hoogendoorn, A., Kleiboer, A., Mira, A., Mackinnon, A., Meyer, B., Botella, C., Littlewood, E.*, et al.* (2017). Efficacy of Self-guided Internet-Based Cognitive Behavioral Therapy in the Treatment of Depressive Symptoms: A Meta-analysis of Individual Participant Data. JAMA Psychiatry *74*, 351-359.

Kiecolt-Glaser, J.K., Bennett, J.M., Andridge, R., Peng, J., Shapiro, C.L., Malarkey, W.B., Emery, C.F., Layman, R., Mrozek, E.E., and Glaser, R. (2014). Yoga's impact on inflammation, mood, and fatigue in breast cancer survivors: a randomized controlled trial. J Clin Oncol *32*, 1040-1049.

Klein, J.P., Berger, T., Schroder, J., Spath, C., Meyer, B., Caspar, F., Lutz, W., Arndt, A., Greiner, W., Grafe, V.*, et al.* (2016). Effects of a Psychological Internet Intervention in the Treatment of Mild to Moderate Depressive Symptoms: Results of the EVIDENT Study, a Randomized Controlled Trial. Psychother Psychosom *85*, 218-228.

Klein, J.P., Berger, T., Schroder, J., Spath, C., Meyer, B., Caspar, F., Lutz, W., Greiner, W., Hautzinger, M., Rose, M.*, et al.* (2013). The EVIDENT-trial: protocol and rationale of a multicenter randomized controlled trial testing the effectiveness of an online-based psychological intervention. BMC Psychiatry *13*, 239.

Kohler, O., Benros, M.E., Nordentoft, M., Farkouh, M.E., Iyengar, R.L., Mors, O., and Krogh, J. (2014). Effect of anti-inflammatory treatment on depression, depressive symptoms, and adverse effects: a systematic review and meta-analysis of randomized clinical trials. JAMA Psychiatry *71*, 1381-1391.

Kreienberg, R., Albert, U., Follmann, M., Kopp, I., Kühn, T., and Wöckel, A. (2013). Interdisziplinäre S3-Leitlinie für die Diagnostik, Therapie und Nachsorge des Mammakarzinoms. Senologie-Zeitschrift für Mammadiagnostik und-therapie *10*, 164-192.

Kuemmel, S., and Schmidt, M. (2016). AGO Guidelines Breast Version 2016.1D: Komplementäre Therapien Hormontherapie „Survivorship“ (Rezidiv-Prävention). https://wwwago-onlinede/fileadmin/downloads/leitlinien/mamma/Maerz2016/de/2016D%2024_Komplementaere%20Therapie.pdf [Stand: 18052017].

Ladwig, I., Rief, W., and Nestoriuc, Y. (2014). Welche Risiken und Nebenwirkungen hat Psychotherapie? - Entwicklung des Inventars zur Erfassung Negativer Effekte von Psychotherapie (INEP). Verhaltenstherapie *24*, 252-263.

Leonard, B.E. (2017). Inflammation and depression: a causal or coincidental link to the pathophysiology? Acta Neuropsychiatr, 1-16.

Littell, R.C., Milliken, G.A., Stroup, W.W., Wolfinger, R.D., and Schabenberger, O. (2006). SAS® System for Mixed Models, Second Edition (Cary, NC: SAS Institute Inc.).

Louati, K., and Berenbaum, F. (2015). Fatigue in chronic inflammation - a link to pain pathways. Arthritis Res Ther *17*, 254.

Maass, S.W., Roorda, C., Berendsen, A.J., Verhaak, P.F., and de Bock, G.H. (2015). The prevalence of long-term symptoms of depression and anxiety after breast cancer treatment: A systematic review. Maturitas *82*, 100-108.

McGregor, B.A., and Antoni, M.H. (2009). Psychological intervention and health outcomes among women treated for breast cancer: a review of stress pathways and biological mediators. Brain Behav Immun *23*, 159-166.

Meyer, B., Berger, T., Caspar, F., Beevers, C.G., Andersson, G., and Weiss, M. (2009). Effectiveness of a novel integrative online treatment for depression (Deprexis): randomized controlled trial. J Med Internet Res *11*, e15.

Meyer, B., Bierbrodt, J., Schröder, J., Berger, T., Beevers, C.G., Weiss, M., Jacob, G., Späth, C., Andersson, G., Lutz, W.*, et al.* (2015). Effects of an Internet intervention (Deprexis) on severe depression symptoms: Randomized controlled trial. Internet Interventions *2*, 48-59.

Meyer, B., Weiss, M., Holtkamp, M., Arnold, S., Brückner, K., Schröder, J., . . . Nestoriuc, Y. (2017). Protocol for the ENCODE trial: evaluating a novel online depression intervention for persons with epilepsy. BMC Psychiatry *17(1),* 55.

Moreira, F.P., Cardoso Tde, A., Mondin, T.C., Souza, L.D., Silva, R., Jansen, K., Oses, J.P., and Wiener, C.D. (2015). The effect of proinflammatory cytokines in Cognitive Behavioral Therapy. J Neuroimmunol *285*, 143-146.

Moritz, S., Schilling, L., Hauschildt, M., Schroder, J., and Treszl, A. (2012). A randomized controlled trial of internet-based therapy in depression. Behav Res Ther *50*, 513-521.

Pierce, B.L., Ballard-Barbash, R., Bernstein, L., Baumgartner, R.N., Neuhouser, M.L., Wener, M.H., Baumgartner, K.B., Gilliland, F.D., Sorensen, B.E., McTiernan, A.*, et al.* (2009). Elevated biomarkers of inflammation are associated with reduced survival among breast cancer patients. J Clin Oncol *27*, 3437-3444.

Porter, K.E. (2016). Statistical Power in Evaluations that Investigate Effects on Multiple Outcomes: A Guide for Researchers (New York, USA: MDRC).

Raison, C.L., Rutherford, R.E., Woolwine, B.J., Shuo, C., Schettler, P., Drake, D.F., Haroon, E., and Miller, A.H. (2013). A randomized controlled trial of the tumor necrosis factor antagonist infliximab for treatment-resistant depression: the role of baseline inflammatory biomarkers. JAMA Psychiatry *70*, 31-41.

Schroder, J., Bruckner, K., Fischer, A., Lindenau, M., Kother, U., Vettorazzi, E., and Moritz, S. (2014). Efficacy of a psychological online intervention for depression in people with epilepsy: a randomized controlled trial. Epilepsia *55*, 2069-2076.

Sheehan, D.V., Lecrubier, Y., Sheehan, K.H., Amorim, P., Janavs, J., Weiller, E., Hergueta, T., Baker, R., and Dunbar, G.C. (1998). The Mini-International Neuropsychiatric Interview (M.I.N.I.): the development and validation of a structured diagnostic psychiatric interview for DSM-IV and ICD-10. J Clin Psychiatry *59 Suppl 20*, 22-33;quiz 34-57.

Staud, R. (2015). Cytokine and immune system abnormalities in fibromyalgia and other central sensitivity syndromes. Curr Rheumatol Rev *11*, 109-115.

Stokes, M., Davis, C., and Koch, G. (2012). Categorical Data Analysis Using SAS®, Third Edition (Cary, NC: SAS Institute).

Von Ah, D., and Kang, D.H. (2008). Correlates of mood disturbance in women with breast cancer: patterns over time. J Adv Nurs *61*, 676-689.

Walsh, E., Eisenlohr-Moul, T., and Baer, R. (2016). Brief mindfulness training reduces salivary IL-6 and TNF-alpha in young women with depressive symptomatology. J Consult Clin Psychol *84*, 887-897.

Zill, J. M., Meyer, B., Topp, J., Daubmann, A., Härter, M., & Dirmaier, J. (2016). Vorvida: study protocol of a randomized controlled trial testing the effectiveness of Internet-based self-help program for the reduction of alcohol consumption for adults. BMC Psychiatry, *16*, 19.

Zwerenz, R., Becker, J., Knickenberg, R. J., Siepmann, M., Hagen, K., & Beutel, M. E. (2017). Online Self-Help as an Add-On to Inpatient Psychotherapy: Efficacy of a New Blended Treatment Approach. Psychotherapy and Psychosomatics *86,* 341-350.
