# Supplementary material for: Results of the Optimune trial: A randomized controlled trial evaluating a novel Internet intervention for breast cancer survivors
Source: PLoS One. 2021 May 7;16(5):e0251276. doi: 10.1371/journal.pone.0251276 (PMC8104369; doi:10.1371/journal.pone.0251276)
Supplement: S1 Table — (DOCX) [file pone.0251276.s004.docx]

**S1 Table.** **PP analysis of primary endpoints**

|  |  | Pre  (T0) |  | Post  (T1) |  | WG Effect Size  Pre-Post | BG Effect Size  Post | Pooled Results  of Fitting Performed Over  Imputed Datasets |  | Wilcoxon  Rank Sum Test |
| --- | --- | --- | --- | --- | --- | --- | --- | --- | --- | --- |
|  |  | Baseline |  | 3 Months |  | WG Change | BG Difference | DV = Post;  IV = Group; Cov = Pre |  | BG |
|  |  | mean | SD | mean | SD | Cohen’s *d* (95% CI) | Cohen’s *d* (95% CI) | *p*-value |  | *p*-value |
| **WHOQOL-BREF total** | IG | 67.02 | 14.16 | 70.38 | 13.84 | 0.24 (0.01-0.47) | 0.34 (0.12-0.56) | 0.002 | T0 | 0.119 |
|  | CG | 65.05 | 12.86 | 65.71 | 13.57 | 0.05 (-0.16-0.26) |  |  | T1 | 0.001 |
| WHOQOL-BREF physical | IG | 65.62 | 17.47 | 72.38 | 14.60 | 0.42 (0.19-0.65) | 0.34 (0.12-0.56) | 0.001 | T0 | 0.361 |
|  | CG | 63.76 | 18.28 | 66.93 | 17.15 | 0.18 (-0.03-0.38) |  |  | T1 | 0.004 |
| WHOQOL-BREF psychological | IG | 62.47 | 16.09 | 69.20 | 14.70 | 0.44 (0.21-0.67) | 0.45 (0.23-0.66) | <0.001 | T0 | 0.151 |
|  | CG | 59.96 | 16.84 | 62.38 | 15.77 | 0.15 (-0.06-0.35) |  |  | T1 | <0.001 |
| WHOQOL-BREF social | IG | 63.03 | 20.34 | 63.63 | 19.34 | 0.03 (-0.20-0.26) | 0.09 (-0.13-0.30) | 0.520 | T0 | 0.450 |
|  | CG | 62.05 | 18.80 | 62.00 | 17.91 | 0.00 (-0.21-0.20) |  |  | T1 | 0.460 |
| WHOQOL-BREF environment | IG | 76.76 | 14.10 | 79.10 | 13.28 | 0.17 (-0.06-0.40) | 0.32 (0.10-0.54) | 0.015 | T0 | 0.152 |
|  | CG | 74.84 | 13.04 | 74.90 | 13.10 | 0.00 (-0.20-0.21) |  |  | T1 | 0.001 |
| **IPAQ total MET** | IG | 3777 | 2948 | 3992 | 2570 | 0.08 (-0.15-0.30) | 0.31 (0.10-0.53) | 0.042 | T0 | 0.173 |
|  | CG | 3304 | 2616 | 3180 | 2584 | -0.05 (-0.25-0.16) |  |  | T1 | <0.001 |
| IPAQ anaerobic MET | IG | 1085 | 1286 | 1061 | 967 | -0.02 (-0.25-0.20) | 0.13 (-0.08-0.35) | 0.246 | T0 | 0.678 |
|  | CG | 1079 | 1320 | 925 | 1044 | -0.13 (-0.33-0.08) |  |  | T1 | 0.042 |
| IPAQ aerobic MET | IG | 1074 | 1232 | 1321 | 1479 | 0.18 (-0.04-0.41) | 0.31 (0.10-0.53) | 0.028 | T0 | 0.055 |
|  | CG | 853 | 1119 | 903 | 1206 | 0.04 (-0.16-0.25) |  |  | T1 | <0.001 |
| IPAQ walk | IG | 1507 | 1367 | 1575 | 1270 | 0.05 (-0.17-0.28) | 0.22 (0.01-0.44) | 0.403 | T0 | 0.016 |
|  | CG | 1129 | 1044 | 1295 | 1227 | 0.15 (-0.06-0.35) |  |  | T1 | 0.007 |
| IPAQ sit | IG | 2450 | 1191 | 2376 | 1084 | 0.07 (-0.16-0.29) | 0.22 (0.00-0.43) | 0.184 | T0 | 0.275 |
|  | CG | 2614 | 1190 | 2618 | 1153 | 0.00 (-0.20-0.21) |  |  | T1 | 0.038 |
| **FQQ total** | IG | 2.10 | 0.39 | 2.17 | 0.31 | 0.20 (-0.03-0.42) | 0.39 (0.17-0.61) | <0.001 | T0 | 0.424 |
|  | CG | 2.08 | 0.38 | 2.04 | 0.33 | -0.10 (-0.31-0.11) |  |  | T1 | <0.001 |
| FQQ healthy | IG | 1.88 | 0.47 | 2.03 | 0.46 | 0.31 (0.08-0.54) | 0.38 (0.17-0.60) | <0.001 | T0 | 0.317 |
|  | CG | 1.83 | 0.48 | 1.84 | 0.49 | 0.02 (-0.18-0.23) |  |  | T1 | 0.001 |
| FQQ unhealthy | IG | 0.69 | 0.47 | 0.56 | 0.39 | 0.30 (0.08-0.53-) | 0.14 (-0.08-0.35) | 0.062 | T0 | 0.945 |
|  | CG | 0.68 | 0.45 | 0.61 | 0.40 | 0.16 (-0,04-0,37) |  |  | T1 | 0.218 |

Note. Results of PP analysis of primary endpoints. WHOQOL-BREF (World Health Organisation Quality of Life questionnaire), IPAQ (International Physical Activity questionnaire), anaerobic (time spent with anaerobic, strenuous activity), aerobic (time spent with aerobic activity), walk (time spent walking), sit (time spent sitting), MET (metabolic equivalent task, in minutes per week), IG (intervention group), CG (control group), SD (standard deviation), WG (within group), BG (between group), DV (dependent variable), IV (independent variable), COV (Covariate), CI (confidence interval), Pre (time point of baseline, before start of intervention, T0), Post (time point of 3 months after start of intervention, T1)
