# Supplementary material for: Results of the Optimune trial: A randomized controlled trial evaluating a novel Internet intervention for breast cancer survivors
Source: PLoS One. 2021 May 7;16(5):e0251276. doi: 10.1371/journal.pone.0251276 (PMC8104369; doi:10.1371/journal.pone.0251276)
Supplement: S2 Table — (DOCX) [file pone.0251276.s005.docx]

**S2 Table.** **PP analysis of secondary endpoints**

|  |  | Pre  (T0) |  | Post  (T1) |  | WG Effect Size  Pre-Post | BG Effect Size  Post | Pooled Results  of Fitting Performed Over  Imputed Datasets |  | Wilcoxon  Rank Sum Test |
| --- | --- | --- | --- | --- | --- | --- | --- | --- | --- | --- |
|  |  | Baseline |  | 3 Months |  | WG Change | BG Difference | DV = Post; IV = Group; Cov = Pre |  | BG |
|  |  | mean | SD | mean | SD | Cohen’s *d* (95% CI) | Cohen’s *d* (95% CI) | *p*-value |  | *p*-value |
| ISI | IG | 11.76 | 6.00 | 9.74 | 5.72 | 0.34 (0.12-0.57) | 0.36 (0.14-0.58) | <0.001 | T0 | 0.765 |
|  | CG | 12.08 | 5.72 | 11.77 | 5.58 | 0.05 (-0.15-0.26) |  |  | T1 | 0.002 |
| BFI | IG | 4.31 | 2.30 | 3.97 | 2.18 | 0.16 (-0.07-0.38) | 0.29 (0.07-0.50) | 0.029 | T0 | 0.099 |
|  | CG | 4.71 | 2.24 | 4.60 | 2.28 | 0.05 (-0.16-0.25) |  |  | T1 | 0.009 |
| IES-R | IG | 1.68 | 0.92 | 1.40 | 0.86 | 0.32 (0.09-0.55) | 0.05 (-0.17-0.27) | 0.044 | T0 | 0.252 |
|  | CG | 1.59 | 1.01 | 1.45 | 0.94 | 0.14 (-0.06-0.35) |  |  | T1 | 0.799 |
| PHQ-9 | IG | 8.66 | 4.96 | 6.82 | 4.34 | 0.40 (0.17-0.62) | 0.34 (0.12-0.56) | <0.001 | T0 | 0.207 |
|  | CG | 9.30 | 5.05 | 8.37 | 4.78 | 0.19 (-0.02-0.39) |  |  | T1 | 0.002 |
| GAD-7 | IG | 7.67 | 4.66 | 6.68 | 4.09 | 0.23 (0.00-0.45) | 0.12 (-0.09-0.34) | 0.083 | T0 | 0.904 |
|  | CG | 7.62 | 4.69 | 7.21 | 4.40 | 0.09 (-0.12-0.30) |  |  | T1 | 0.275 |
| PA-F12 | IG | 35.96 | 9.15 | 33.73 | 9.17 | 0.24 (0.02-0.47) | 0.14 (-0.08-0.35) | 0.037 | T0 | 0.740 |
|  | CG | 35.87 | 9.53 | 35.03 | 9.75 | 0.09 (-0.12-0.29) |  |  | T1 | 0.299 |

*Note.* Results of PP analysis of secondary endpoints. ISI (Insomnia Severity Index), BFI (Brief Fatigue Inventory, cancer-specific), IES-R (Intrusion scale of the Impact of Event Scale Revised), PHQ-9 (Patient Health Questionnaire-9), GAD-7 (General Anxiety Disorder-7), PA-F12 (Fear of Progression), IG (intervention group), CG (control group), SD (standard deviation), WG (within group), BG (between group), DV (dependent variable), IV (independent variable), COV (Covariate), CI (confidence interval), Pre (time point of baseline, before start of intervention, T0), Post (time point of 3 months after start of intervention, T1).
