# Supplementary material for: Results of the Optimune trial: A randomized controlled trial evaluating a novel Internet intervention for breast cancer survivors
Source: PLoS One. 2021 May 7;16(5):e0251276. doi: 10.1371/journal.pone.0251276 (PMC8104369; doi:10.1371/journal.pone.0251276)
Supplement: S3 Table — Changes between 3 and 6 months (CG accessed intervention after 3 months). (DOCX) [file pone.0251276.s006.docx]

**S3 Table.** **Changes between 3 and 6 months (CG accessed intervention after 3 months)**

|  |  |  | 3 months |  | 6 months |  | WG change, | Wilcoxon signed rank test | 6 months |  | Wilcoxon rank sum test |
| --- | --- | --- | --- | --- | --- | --- | --- | --- | --- | --- | --- |
|  |  |  | mean | SD | mean | SD | Cohen's d | WG from T1 to T2 | BG difference, |  | BG @ pre & post |
|  |  |  |  |  |  |  |  | p-value | Cohen's d |  | p-value |
| Primary | **WHOQOL-BREF_total** | IG | 72.31 | 13.37 | 72.20 | 13.88 | 0.01 | 0.863 | 0.28 | T1 | <0.001 |
| outcomes |  | CG | 65.69 | 13.59 | 68.38 | 13.23 | 0.20 | <0.001 |  | T2 | 0.006 |
|  | WHOQOL-BREF_physical | IG | 74.07 | 14.80 | 74.88 | 16.07 | 0.05 | 0.262 | 0.35 | T1 | <0.001 |
|  |  | CG | 65.99 | 17.64 | 69.15 | 16.42 | 0.19 | <0.001 |  | T2 | 0.001 |
|  | WHOQOL-BREF_psychological | IG | 70.16 | 15.36 | 69.98 | 16.82 | 0.01 | 0.713 | 0.23 | T1 | <0.001 |
|  |  | CG | 61.44 | 16.09 | 66.30 | 15.84 | 0.30 | <0.001 |  | T2 | 0.014 |
|  | WHOQOL-BREF_social | IG | 66.12 | 19.75 | 65.65 | 19.09 | 0.02 | 0.569 | 0.17 | T1 | 0.064 |
|  |  | CG | 62.12 | 18.55 | 62.61 | 17.72 | 0.03 | 0.379 |  | T2 | 0.110 |
|  | WHOQOL-BREF_environment | IG | 79.83 | 13.98 | 80.12 | 12.80 | 0.02 | 0.724 | 0.28 | T1 | <0.001 |
|  |  | CG | 74.28 | 13.49 | 76.50 | 13.16 | 0.17 | <0.001 |  | T2 | 0.013 |
|  | **IPAQ_total_MET** | IG | 3985 | 2627 | 3454 | 2394 | 0.21 | 0.052 | 0.11 | T1 | 0.001 |
|  |  | CG | 3204 | 2571 | 3200 | 2085 | 0.00 | 0.701 |  | T2 | 0.490 |
|  | IPAQ_anaerob_MET | IG | 1092 | 1040 | 1077 | 951 | 0.02 | 0.905 | 0.03 | T1 | 0.033 |
|  |  | CG | 927 | 1106 | 1050 | 974 | 0.12 | 0.400 |  | T2 | 0.519 |
|  | IPAQ_aerob_MET | IG | 1378 | 1607 | 868 | 910 | 0.39 | 0.015 | 0.02 | T1 | 0.001 |
|  |  | CG | 891 | 1267 | 888 | 959 | 0.00 | 0.175 |  | T2 | 0.905 |
|  | IPAQ_walk_MET | IG | 1534 | 1289 | 1411 | 1282 | 0.10 | 0.118 | 0.15 | T1 | 0.058 |
|  |  | CG | 1329 | 1298 | 1227 | 1150 | 0.08 | 0.529 |  | T2 | 0.248 |
|  | IPAQ_sit | IG | 2305 | 1041 | 2386 | 1035 | 0.08 | 0.148 | 0.14 | T1 | 0.011 |
|  |  | CG | 2624 | 1151 | 2530 | 1061 | 0.08 | 0.335 |  | T2 | 0.271 |
|  | **FQQ_total** | IG | 2.20 | 0.29 | 2.25 | 0.31 | 0.18 | 0.006 | 0.12 | T1 | <0.001 |
|  |  | CG | 2.04 | 0.33 | 2.21 | 0.35 | 0.51 | <0.001 |  | T2 | 0.425 |
|  | FQQ_healthy | IG | 2.06 | 0.45 | 2.04 | 0.42 | 0.04 | 0.288 | 0.10 | T1 | <0.001 |
|  |  | CG | 1.84 | 0.50 | 2.00 | 0.45 | 0.34 | <0.001 |  | T2 | 0.374 |
|  | FQQ_unhealthy | IG | 0.50 | 0.36 | 0.54 | 0.38 | 0.09 | 0.109 | 0.08 | T1 | 0.018 |
|  |  | CG | 0.61 | 0.40 | 0.57 | 0.40 | 0.10 | 0.016 |  | T2 | 0.555 |
| Secondary | ISI | IG | 9.41 | 5.94 | 8.92 | 5.64 | 0.08 | 0.268 | 0.34 | T1 | <0.001 |
| outcomes |  | CG | 11.88 | 5.58 | 10.70 | 4.92 | 0.22 | 0.001 |  | T2 | 0.001 |
|  | BFI | IG | 3.73 | 2.13 | 3.24 | 2.12 | 0.23 | 0.006 | 0.42 | T1 | 0.001 |
|  |  | CG | 4.61 | 2.27 | 4.15 | 2.22 | 0.21 | 0.001 |  | T2 | <0.001 |
|  | IES-R | IG | 1.35 | 0.88 | 1.34 | 0.82 | 0.00 | 0.793 | 0.06 | T1 | 0.384 |
|  |  | CG | 1.45 | 0.94 | 1.29 | 0.88 | 0.18 | <0.001 |  | T2 | 0.438 |
|  | PHQ-9 | IG | 6.26 | 4.16 | 6.33 | 4.27 | 0.02 | 0.988 | 0.30 | T1 | <0.001 |
|  |  | CG | 8.41 | 4.77 | 7.63 | 4.41 | 0.17 | 0.003 |  | T2 | 0.010 |
|  | GAD-7 | IG | 6.29 | 4.09 | 5.98 | 4.03 | 0.08 | 0.159 | 0.13 | T1 | 0.050 |
|  |  | CG | 7.23 | 4.39 | 6.56 | 4.45 | 0.15 | 0.001 |  | T2 | 0.337 |
|  | PA-F12 | IG | 33.11 | 9.25 | 32.57 | 8.85 | 0.06 | 0.140 | 0.08 | T1 | 0.103 |
|  |  | CG | 35.04 | 9.75 | 33.29 | 9.30 | 0.18 | <0.001 |  | T2 | 0.701 |

Note. Results of follow up analysis of primary and secondary endpoints. WHOQOL-BREF (World Health Organisation Quality of Life questionnaire), IPAQ (International Physical Activity questionnaire), anaerobic (time spent with anaerobic, strenuous activity), aerobic (time spent with aerobic activity), walk (time spent walking), sit (time spent sitting), MET (metabolic equivalent task, in minutes per week), ISI (Insomnia Severity Index), BFI (Brief Fatigue Inventory, cancer-specific), IES-R (Intrusion scale of the Impact of Event Scale Revised), PHQ-9 (Patient Health Questionnaire-9), GAD-7 (General Anxiety Disorder-7), PA-F12 (Fear of Progression), IG (intervention group), CG (control group), SD (standard deviation), WG (within group), BG (between group), DV (dependent variable), IV (independent variable), COV (Covariate), CI (confidence interval), Pre (time point of 3 months after start of intervention, T1), Post (time point of 6 months after start of intervention, T2).
